# Genome-wide association study of subclinical interstitial lung disease in MESA

**DOI:** 10.1186/s12931-017-0581-2

**Published:** 2017-05-18

**Authors:** Ani Manichaikul, Xin-Qun Wang, Li Sun, Josée Dupuis, Alain C. Borczuk, Jennifer N. Nguyen, Ganesh Raghu, Eric A. Hoffman, Suna Onengut-Gumuscu, Emily A. Farber, Joel D. Kaufman, Dan Rabinowitz, Karen D. Hinckley Stukovsky, Steven M. Kawut, Gary M. Hunninghake, George R. Washko, George T. O’Connor, Stephen S. Rich, R. Graham Barr, David J. Lederer

**Affiliations:** 1 Center for Public Health Genomics, University of Virginia, Charlottesville, VA USA; 2 Department of Public Health Sciences, Biostatistics Section, University of Virginia, Charlottesville, VA USA; 3 Department of Medicine, College of Physicians and Surgeons, Columbia University, New York, NY USA; 4 Department of Biostatistics, Boston University School of Public Health, Boston, MA USA; 5 The National Heart, Lung, and Blood Institute’s Framingham Heart Study, Framingham, MA USA; 6 Department of Pathology, Weill Cornell Medicine, New York, NY USA; 7 University of Washington Center for Interstitial Lung Diseases, Seattle, WA USA; 8 Department of Radiology, University of Iowa Carver College of Medicine, Iowa City, IA, USA; 9 Departmenst of Environmental & Occupational Health Sciences, Medicine, and Epidemiology, University of Washington, Seattle, WA USA; 10 Department of Statistics, Columbia University, New York, NY USA; 11 Department of Biostatistics, University of Washington, Seattle, WA USA; 12 Department of Medicine and Center for Clinical Epidemiology and Biostatistics, Perelman School of Medicine, University of Pennsylvania, Philadelphia, PA USA; 13 Division of Pulmonary and Critical Care Medicine, Department of Medicine, Brigham and Women’s Hospital, Boston, MA USA; 14 Pulmonary Center, Department of Medicine, Boston University School of Medicine, Boston, MA USA; 15 Department of Epidemiology, Mailman School of Public Health, Columbia University, New York, NY USA; 16 Center for Public Health Genomics, University of Virginia School of Medicine, West Complex Room 6115, Charlottesville, VA 22903 USA

**Keywords:** Interstitial lung disease, Genetics, Genome-wide association study, Epidemiology

## Abstract

**Background:**

We conducted a genome-wide association study (GWAS) of subclinical interstitial lung disease (ILD), defined as high attenuation areas (HAA) on CT, in the population-based Multi-Ethnic Study of Atherosclerosis Study.

**Methods:**

We measured the percentage of high attenuation areas (HAA) in the lung fields on cardiac CT scan defined as voxels with CT attenuation values between -600 and -250 HU. Genetic analyses were performed in MESA combined across race/ethnic groups: non-Hispanic White (*n* = 2,434), African American (*n* = 2,470), Hispanic (*n* = 2,065) and Chinese (*n* = 702), as well as stratified by race/ethnicity.

**Results:**

Among 7,671 participants, regions at genome-wide significance were identified for basilar peel-core ratio of HAA in *FLJ35282* downstream of *ANRIL* (rs7852363, *P* = 2.1x10^−9^) and within introns of *SNAI3-AS1* (rs140142658, *P* = 9.6x10^−9^) and *D21S2088E* (rs3079677, *P* = 2.3x10^−8^)*.* Within race/ethnic groups, 18 additional loci were identified at genome-wide significance, including genes related to development (*FOXP4*), cell adhesion (*ALCAM*) and glycosylation (*GNPDA2*, *GYPC*, *GFPT1* and *FUT10*). Among these loci, SNP rs6844387 near *GNPDA2* demonstrated nominal evidence of replication in analysis of *n* = 1,959 participants from the Framingham Heart Study (*P* = 0.029). *FOXP4* region SNP rs2894439 demonstrated evidence of validation in analysis of *n* = 228 White ILD cases from the Columbia ILD Study compared to race/ethnicity-matched controls from MESA (one-sided *P* = 0.007). In lung tissue from 15 adults with idiopathic pulmonary fibrosis compared to 15 adults without lung disease. *ANRIL* (*P* = 0.001), *ALCAM* (*P* = 0.03) and *FOXP4* (*P* = 0.046) were differentially expressed.

**Conclusions:**

Our results suggest novel roles for protein glycosylation and cell cycle disinhibition by long non-coding RNA in the pathogenesis of ILD.

**Electronic supplementary material:**

The online version of this article (doi:10.1186/s12931-017-0581-2) contains supplementary material, which is available to authorized users.

## Background

The interstitial lung diseases (ILDs) are a family of non-infectious, non-malignant lung diseases characterized by alveolar injury, inflammation, and fibrosis. Idiopathic pulmonary fibrosis (IPF), the most common idiopathic ILD, has a median survival time of 3.8 years and affects nearly 1 out of 200 older adults in the United States [1]. Previous genome-wide association studies (GWAS) for IPF have demonstrated the utility of genetic approaches for discovering novel biological pathways involved in the pathogenesis of ILD in studies of up to ~1,600 cases and ~4,700 controls [2, 3]. Variation in the promoter of *MUC5B* was identified as a risk factor for clinically evident sporadic and familial ILD [4] and is associated with the presence of interstitial lung abnormalities (ILAs), a qualitative subclinical ILD phenotype, in the Framingham Heart Study (FHS) [5]. Other genes implicated in the development of ILD include *SFTPA2, SFTPC, ELMOD2, TERT, TERC, FAM13A*, *DSP*, *OBFC1*, *DPP9, TOLLIP, ATP11A*, and *SPPL2C* [2, 3].

The study of early, subclinical disease before advanced parenchymal changes have occurred could lead to the identification of novel biological pathways involved in the pathogenesis of ILD at a stage more amenable for intervention. High attenuation areas (HAA), defined as the percentage of lung voxels in the range of -600 – −250 Hounsfield units, correspond to the CT attenuation values observed in areas visually described as having ground-glass and reticular attenuation [6]. In the Multi-Ethnic Study of Atherosclerosis (MESA), we have shown that greater HAA is associated with cigarette smoking, lower forced vital capacity, reduced exercise capacity, higher serum levels of matrix metalloproteinase-7 (MMP-7) and interleukin-6, a higher prevalence of interstitial lung abnormalities (ILAs) at 9.5 years follow-up, and a higher risk of death [6, 7].

Under the hypothesis that high throughput genetic association analysis could identify novel pathophysiologic pathways to explain the occurrence of HAA and ILD, we conducted a GWAS for percent HAA on cardiac CT in the MESA Study. We further sought replication of identified SNPs for percent HAA in participants of European ancestry from the FHS, validation in analysis of ILD cases from the Columbia ILD Study compared to matched controls selected from among the MESA participants, and performed differential gene expression analysis for comparing mRNA from lung tissue of IPF cases versus controls.

## Methods

### Study participants

MESA is a population-based longitudinal study of subclinical cardiovascular disease [8]. Between 2000 and 2002, MESA recruited 6,814 men and women 45–84 years of age from six US sites who were free of clinical cardiovascular disease. The MESA Family Study recruited an additional 1,595 African-American and Hispanic family members 45–84 years of age specifically for genetic analysis, and the MESA Air Pollution Study recruited an additional 257 participants [9]. Participants who did not consent to genetic analyses or who had no usable genetic material were excluded, resulting in a combined sample of 7,671 participants comprising the MESA SNP Health Association Resource (SHARe) sample, which has been previously described [10].

We sought replication of selected SNPs from the race/ethnic specific GWAS of MESA Whites in the Framingham Heart Study (FHS), an independent population-based cohort. See *Online Supplement* for details.

### Percent high attenuation areas

The MESA Lung Fibrosis Study was developed to determine the percent HAA for all participants on the lung fields of cardiac CT scans, which were acquired under a standardized protocol [6, 11]. For discovery analyses in MESA as well as replication in FHS, percent HAA was defined at lung regions between -600 and -250 Hounsfield Units (HU), with basilar percent HAA quantified as the percent HAA in the caudal 1/3^rd^ of the imaged lung. In MESA, basilar peel-core ratio of HAA (henceforth termed “basilar peel-core ratio”) computed as the percent HAA in the peel region (outer 20 mm) divided by that in the core region for the caudal 1/3^rd^ of the imaged lung.

### Genotyping

Genome-wide genotyping was performed for MESA and FHS participants followed by imputation the 1000 Genomes reference panel [12]. Additional details are provided in the *Online Supplement*.

### Genetic association analysis

Within the MESA Lung Fibrosis study, we completed GWAS analyses of percent HAA, basilar percent HAA, and basilar peel-core ratio. Race/ethnic-specific analyses were performed in linear regression models with adjustment for age, sex, study site, principal components (PCs) of ancestry, CT scanner, tube current, breath artifacts, height, weight, cigarettes per day (for current smokers only) and pack-years of smoking. We did not adjust for dichotomous smoking exposures (current/former/never smoking) because this information was already captured by the continuous measures of smoking exposure. Results were combined by fixed-effect meta-analyses across all four MESA race/ethnic groups.

We examined genomic control values of all GWAS for evidence of residual population stratification, undetected family structure, or other sources of inflation in type I error. Single SNP association results that attained a threshold of *P* < 5.0 x 10^−8^ were considered genome-wide significant. Those SNPs demonstrating genome-wide significant association in discovery GWAS of percent HAA and basilar percent HAA in MESA were examined for evidence of replication with adjustment for the same covariates in FHS. Additional details provided in the *Online Supplement*.

### Validation with ILD cases

Three hundred sixteen ILD cases (108 of these were IPF confirmed) were recruited between 2007 and 2011 through the Columbia ILD Study, an NIH-funded prospective cohort study at Columbia University Medical Center. For selected SNPs, we performed genetic analysis with these ILD cases compared to MESA controls matched on race/ethnicity. See *Online Supplement* for details.

### Gene expression analyses

We measured mRNA expression of four of the genes we identified (*ALCAM*, *FOXP4*, *CDKN2B*, and *ANRIL*, along with the reference gene *GAPDH*) in OCT-embedded fresh frozen lung tissue obtained from 15 adults with IPF and a histologic usual interstitial pneumonia pattern (UIP) and 15 adults without lung disease stored in the Columbia University Pathology Tissue Bank. See *Online Supplement* for details.

## Results

### Characteristics of the MESA lung fibrosis participants

MESA Lung Fibrosis participants [10] represent a relatively healthy cohort ages 39–91 years, 54% of whom were female, with race/ethnic distribution consisting of 32% non-Hispanic White (henceforth termed “White”), 32% African American, 27% Hispanic, and 9% Chinese. Smoking history varied considerably across race/ethnic groups: 56% of Whites, 53% of African Americans, 45% of Hispanics, and 25% of Chinese participants had ever smoked.

The median percent HAA ranged from 3.9% in Whites to 4.5% in Hispanic and Chinese, and median basilar peel-core ratio ranged from 0.89 in Chinese to 0.96 in Whites. Percent HAA and basilar peel-core ratio were relatively distinct from one another, with correlations for ranging from 0.01 to 0.17 within race/ethnic groups (Table 1).

**Table 1:** Characteristics of participants in the MESA Fibrosis Lung/SHARe Study

	White	African American	Hispanic	Chinese
Participant characteristics^a^				
No. subjects	2434	2470	2065	702
Women	1267 (52.1)	1381 (55.9)	1115 (54.0)	355 (50.6)
Age, years	63 [54, 71]	60 [53, 68]	60 [53, 68]	63 [54, 71]
Height, cm	168.7 [161.7, 176.2]	168.0 [161.1, 175.7]	161.1 [155.3, 169.0]	161.6 [154.9, 168.0]
Weight, lbs	172.4 [148.0, 198.5]	186.0 [161.0, 214.2]	168.0 [146.5, 190.5]	136.2 [121.3, 152.5]
Weight > 220 (yes/no)	302 (12.4)	519 (21.0)	173 (8.4)	0 (0)
BMI, kg/m^2^	27.1 [24.2, 30.4]	29.4 [26.1, 33.7]	28.6 [26.0, 32.2]	23.7 [21.7, 26.0]
Diabetes (yes/no)	284 (11.7)	384 (15.6)	333 (16.1)	117 (16.7)
Hypertension (yes/no)	935 (38.4)	1473 (59.6)	864 (41.8)	263 (37.5)
Ever-smoke (yes/no)	1345 (55.3)	1297 (52.5)	927 (44.9)	176 (25.1)
Current-smoke (yes/no)	269 (11.4)	471 (19.2)	280 (13.7)	39 (5.6)
Pack-years of smoking^b^	18.8 [6.5, 37.5]	15.0 [6.5, 28.0]	8.8 [3.0, 22.2]	13.7 [4.4, 26.3]
Cigarettes per day^b^	15 [9, 20]	10 [5, 20]	6 [2,13.3]	10 [7, 15]
Asthma (yes/no)	251 (10.3)	280 (11.3)	219 (10.6)	42 (6.0)
Measures of HAA				
No. subjects	2434	2470	2065	702
Percent HAA	3.9 [3.3, 4.8]	4.3 [3.6, 5.4]	4.5 [3.7, 5.8]	4.5 [3.8, 6.1]
Basilar percent HAA	4.3 [3.4, 5.7]	4.9 [3.8, 6.7]	5.1 [4.0, 7.2]	5.0 [3.8, 7.6]
Basilar peel-core ratio of HAA	0.96 [0.84, 1.12]	0.91 [0.80, 1.06]	0.91 [0.80, 1.04]	0.89 [0.78, 1.01]
Correlation of HAA traits^c^				
Percent HAA:Basilar percent HAA	0.944	0.927	0.944	0.961
Percent HAA:Basilar peel-core ratio	0.164	0.014	0.099	0.173
Basilar percent HAA:Basilar peel-core ratio	0.281	0.161	0.222	0.216
Pulmonary function^a^				
No. subjects	1354	917	1012	562
FEV_1_ (mL)	2515 [2036, 3086]	2130 [1726, 2608]	2362 [1960, 2848]	2167 [1724, 2588]
FVC (mL)	3444 [2808, 4180]	2808 [2321, 3515]	3040 [2525, 3775]	2856 [2313, 3470]
FEV_1_/FVC	0.743 [0.690 0.786]	0.768 [0.708, 0.809]	0.782 [0.740, 0.817]	0.764 [0.715, 0.805]

Data are presented as *n* (%) for binary measures or median [IQR] for continuous measure

^a^Summary statistics are reported for the subset of individuals with data available for at least one of the HAA phenotypes

^b^Pack-years of smoking are reported for ever-smokers only. Cigarettes per day are reported for current smokers only

^c^Correlation of HAA phenotypes is computed using log-transformed phenotype values

### GWAS of HAA phenotypes across race/ethnic groups

**Fig. 1:**
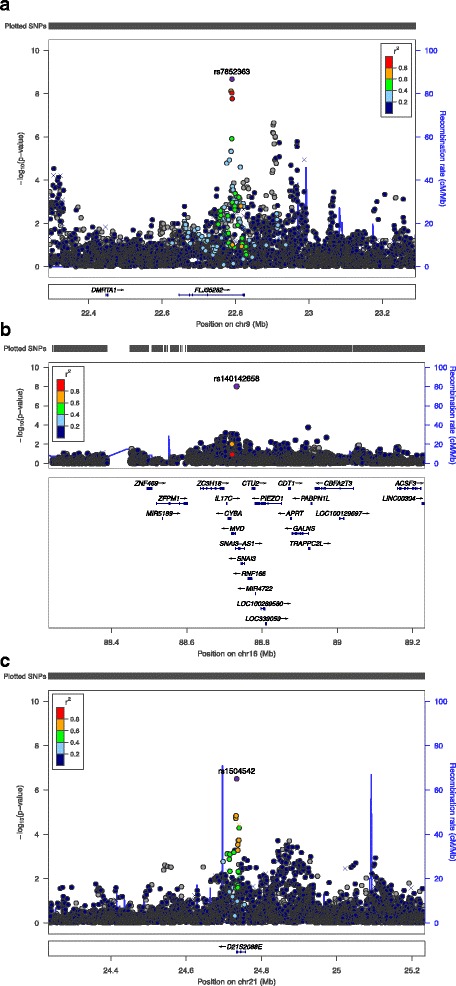
Regional association plots for statistically significant GWAS regions identified in association with basilar peel-core ratio based on meta-analysis to combine results across race/ethnic groups in MESA. **a** Chromosome 9 region including *FLJ35282*, **b** Chromosome 16 region at *SNAI3-AS1* and **c** Chromosome 21 region at *D21S2088E*. All plots are generated using the LocusZoom tool [36] with 1000 Genomes EUR (European ancestry) reference panel used for calculating linkage disequilibrium (LD). The x-axis shows position on the indicated chromosome in megabases (Mb), while the y-axis displays strength of association for each SNP as –log [10] *p*-value (*left*) and recombination rate at each genomic position (*right*). Genotyped SNPs are marked as crosses and imputed SNPs are shown as circles. The SNP indicated in purple is the index SNP, and other SNPs are color coded according to their linkage disequilibrium (R-squared) with the index SNP. LD for (**c**) is shown with respect to the SNP rs1504542 because LD information was not available for rs3079677 in the selected reference panel

In combined analysis across race/ethnic groups, we identified three SNPs at genome-wide significance for basilar peel-core ratio (Table 2, Fig. 1, Additional file 1: Figures S1-S2). The top SNP for the first locus, rs7852363 (*P* = 2.1x10^−9^), lies within the long non-coding RNA gene *FLJ35282*, and about 670 kb downstream of the 9p21 locus near the long non-coding RNA *ANRIL*, widely reported in association with coronary disease risk [13]. The top associated SNP for the second locus, rs140142658 (*P* = 9.6x10^−9^), lies within the first intron of the antisense RNA gene *SNAI3-AS1*. The top associated variant for the third locus, rs3079677 (*P* = 2.3x10^−8^), is a deletion within the last intron of the long non-coding RNA gene *D21S2088E*. For all three of these loci, the effect allele is associated with increased basilar peel-core ratio across each of the MESA race/ethnic groups contributing to the combined result (Additional file 1: Table S1).

**Table 2:** Summary of genome-wide significant results in the GWAS

Trait	Group	SNP ID	Chr	NCBI37 position	Nearest gene(s)	Effect/other allele	Effect allele freq.^a^	Beta	SE	*P*-value
Percent HAA	White	rs6844387	4	44,775,446	*GNPDA2* (upstream)	A/G	0.006	0.634	0.088	5.5E-13
		rs2894439	6	41,480,093	*FOXP4* (upstream)	A/G	0.017	0.202	0.037	3.5E-08
		rs117323377	12	124,715,694	*ZNF664-FAM101A* (intronic)	G/A	0.026	0.149	0.026	1.5E-08
		rs141944608	14	59,455,322	*DAAM1* (upstream)	T/C	0.009	0.311	0.055	1.3E-08
	Chinese	rs79441543	10	3,132,994	*PFKP* (intronic)	C/T	0.048	0.615	0.097	2.8E-10
Basilar percent HAA	White	rs138384996	1	64,724,706	*UBE2U* (downstream)	A/G	0.019	0.438	0.077	1.6E-08
		rs150536895	2	127,254,486	*GYPC* (upstream)	A/G	0.009	0.486	0.088	3.7E-08
		rs146792761	3	103,949,823	*ALCAM* (upstream)	T/G	0.012	0.521	0.092	1.4E-08
	African American	rs145855729	6	36,495,778	*STK38* (intronic)	T/C	0.084	0.126	0.023	4.4E-08
		rs114571830	8	32,973,995	*FUT10* (downstream)	A/C	0.024	0.227	0.041	2.6E-08
		rs149416017	14	55,227,152	*SAMD4A* (intronic)	T/G	0.011	0.411	0.074	3.2E-08
	Chinese	rs74361312	10	107,354,812	*SORCS3* (downstream)	G/C	0.030	0.495	0.090	4.0E-08
Basilar peel-core ratio	White	rs76164182	1	8,291,827	*SLC45A1* (upstream)	C/T	0.018	0.210	0.038	3.2E-08
		rs114801796	1	93,408,101	*FAM69A* (intronic)	C/G	0.017	0.221	0.040	4.4E-08
		rs190432524	5	179,742,263	*GFPT2* (intronic)	T/C	0.009	0.318	0.048	5.4E-11
		rs182717611	18	12,955,146	*SEH1L* (intronic)	T/C	0.020	0.170	0.031	4.7E-08
	African American	rs150377334	8	71,177,444	*NCOA2* (intronic)	A/C	0.009	0.238	0.039	7.6E-10
		rs74315875	14	37,335,713	*SLC25A21* (intronic)	G/C	0.033	0.116	0.019	2.5E-09
	Meta-analysis	rs7852363	9	22,790,485	*FLJ35282* (intronic)	T/C	0.132/0.379/0.162/0.036	0.028	0.005	2.1E-09
		rs140142658	16	88,732,573	*SNAI3-AS1* (intronic)	G/A	0.016/-/0.019/-	0.133	0.023	9.6E-09
		rs3079677	21	24,735,151	*D21S2088E* (intronic)	T/TG	0.430/0.311/0.446/0.578	0.024	0.004	2.3E-08

Results are presented based on the basic model of genetic association, including adjustment for age, sex, study site, principal components of ancestry, CT scanner, tube current, breath artifacts, height, weight, cigarettes per day (for current smokers only) and pack-years. Statistical association for rs6844387 also reached the genome-wide significance threshold for basilar percent HAA, but the association was stronger for percent HAA. ^a^Race/ethnic-specific effect allele frequencies for meta-analysis are shown for White/African American/Hispanic/Chinese, and values omitted in cases that the association results did not pass race/ethnic-specific quality control filters prior to meta-analysis

### GWAS of HAA phenotypes within race/ethnic groups

Among Whites, four loci in or near the genes *GNPDA2*, *FOXP4, ZNF664-FAM101A* and *DAAM1* demonstrated genome-wide significant association with percent HAA (Additional file 1: Figure S3), while three additional loci in or near *UBE2U*, *GYPC*, and *ALCAM* showed genome-wide significant association with basilar HAA (Additional file 1: Figure S4). For basilar peel-core ratio, we also identified genome-wide significance for four loci at *SLC45A1*, *FAM69A*, *GFPT1*, and *SEH1L* (Additional file 1: Figure S5). The identified SNPs were rare/infrequent in Whites with minor allele frequency (MAF) ranging from 0.006 to 0.026 and the minor alleles were all associated with increased HAA, basilar HAA or basilar peel-core ratio (Table 2). Common themes and putative functions of genes identified in Whites include genes that act on glycoproteins and glycosylation (*GNPDA2*, *GYPC*, *GFPT1*), genes important in development (*FOXP4*, *DAAM1*) and cell adhesion (*DAAM1*, *ALCAM*).

Among African Americans, we identified three loci in or near the genes *STK38*, *FUT10* and *SAMD4A* associated with basilar HAA and two loci intronic to the genes *NCOA2* and *SLC25A21* associated with basilar peel-core ratio.

Among Chinese, we identified a locus at genome-wide significance in *PRKP* for percent HAA and near *SORCS3* for basilar percent HAA.

While we noted overlap in the putative function of genes identified in the race/ethnic specific GWAS of HAA, specific variants identified in race/ethnic specific analyses did not generally exhibit consistent effects across race/ethnic groups (Additional file 1: Table S1-S3). SNPs that did show consistent directions of effect across race/ethnic groups include rs190432524 at *GFPT2* for basilar peel-core ratio (Additional file 1: Table S1), and rs6844387 at *GNPDA2* for percent HAA (Additional file 1: Table S2). The results identified for basilar percent HAA appeared to be mostly race/ethnic specific, without shared evidence across groups (Additional file 1: Table S3). Sensitivity analysis demonstrated the main SNP associations reported in Table 2 were largely unchanged after additional adjustment for BMI, waist circumference and diabetes status (Additional file 1: Table S4). Many of the main SNP associations were also robust to covariate adjustment for percent emphysema (Additional file 1: Table S5). However, some SNPs showed attenuated evidence for association after adjustment for percent emphysema resulting from residual association with percent emphysema (Additional file 1: Table S6). In particular, the *FOXP4* SNP rs2894439 demonstrated the strongest evidence of with percent emphysema (*P* = 4.1x10^−6^), although the strength of association with percent emphysema did not reach that seen for percent HAA in primary analysis (Table 2, *P* = 3.5x10^−8^).

### Replication in the Framingham heart study

We examined evidence of replication in *N* = 1959 participants from the Framingham Heart Study (FHS, Additional file 1: Table S7) for the seven SNPs identified in the GWAS of percent HAA and basilar HAA in MESA Whites (shown in Table 2). One SNP rs6844387 near *GNPDA2* demonstrated nominal significance (Additional file 1: Table S8, *P* = 0.029) in this group with the same direction of effect as observed in MESA (Table 2). Meta-analysis across MESA Whites and FHS yielded an attenuated effect size reflecting the smaller effect observed in FHS (combined Beta = 0.419, *P* = 2.3x10^−11^).

### Validation with ILD cases

In race/ethnic specific analysis of Whites, we examined evidence of association for 13 SNPs identified in the GWAS of percent HAA, basilar percent HAA, or basilar peel-core ratio for MESA Whites or in meta-analysis across race/ethnic groups (Table 2). SNP rs2894439 near *FOXP4* demonstrated nominally significant association (Additional file 1: Table S9-S10, one-sided *P* = 0.007) in Whites, with increased frequency in Columbia ILD cases (MAF = 0.048) compared to matched controls free of ILD in MESA (MAF = 0.021). We did not observe any SNPs reaching nominal significance in the combined analysis across race/ethnic groups.

### Examination of SNPs reported in previous GWAS of IPF

We evaluated MESA GWAS results for those SNPs that have been reported in previously published GWAS of IPF [2–4, 14], and identified a total of 33 SNPs of interest as shown in Additional file 1: Table S11. After correction for multiple comparisons (*P* < 0.05/33 comparisons = 0.0015), two intronic SNPs for *TOLLIP* exhibited significant associations with increased percent HAA (Additional file 1: Table S12, rs5743894, *P* = 0.0009) and basilar percent HAA (Additional file 1: Table S13, rs5743894, *P* = 0.0007) in African Americans.

### Gene expression

**Fig. 2:**
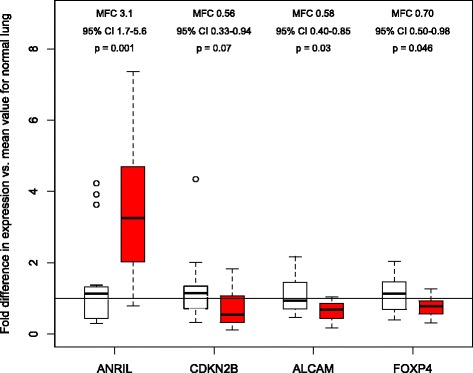
mRNA expression in whole lung tissue for *ANRIL, CDKN2B, ALCAM,* and *FOXP4* from 15 cases with IPF (*red*) and 15 controls (*white*) using quantitative real-time PCR normalized to expression of the reference gene *GADPH*. Results are presented as boxplots of the fold difference in expression in each IPF case or control normalized to the mean expression value among controls. Within each boxplot, the thick horizontal line represents the median fold difference, the ends of the boxplots are placed at the 25^th^ and 75^th^ percentiles (interquartile range), the whiskers extend to 1.5 x the interquartile range, and outliers are represented by open circles. Mean fold change (MFC) was calculated using the ΔΔCt method. P values are from Wilcoxon rank sum tests. The horizontal line at a value of 1 is the mean normalized expression value among controls

**Fig. 3:**
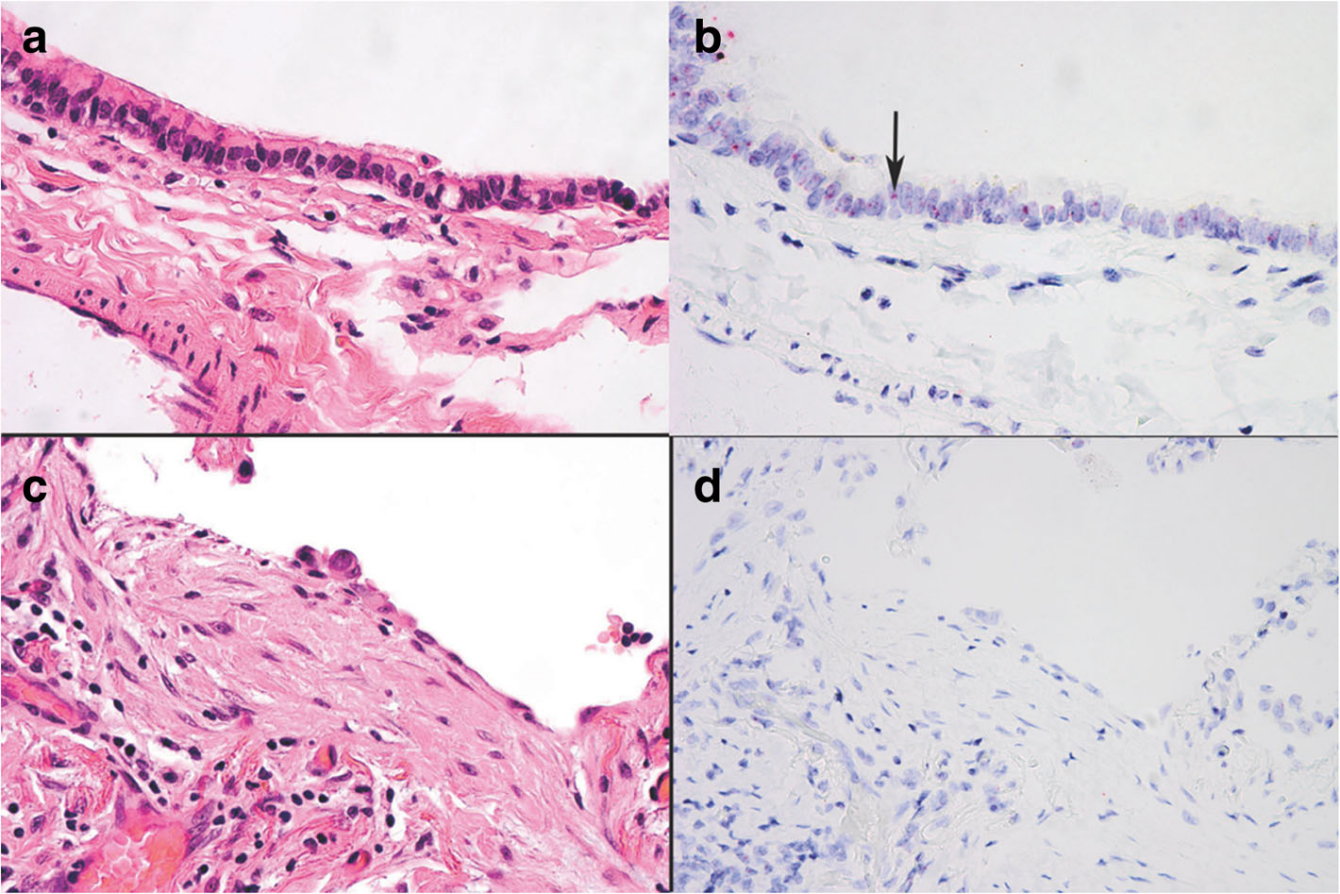
Expression of *ANRIL* (a long non-coding RNA) in UIP lung tissue. H&E stained tissue (left: panels **a** and **c**); *in situ* hybridization of ANRIL transcripts (right: panels **b** and **d**) for airway epithelium (top: panels **a** and **b**) versus fibroblastic foci (bottom: panels **c** and **d**). Note the pink dots (*ANRIL* transcripts) in airway epithelium (**b**) but not in fibroblastic foci (**d**). The photomicrograph is presented at x150 magnification

We selected 4 genes (*ANRIL*, *ALCAM*, *FOXP4*, and *CDKN2B*) for gene expression analyses. We identified a 3-fold increase in *ANRIL* mRNA expression in IPF lung compared to controls (Fig. 2; mean fold change 3.1, 95% CI 1.7 – 5.6, *P* = 0.001). *ANRIL* mRNA localized to airway epithelium in both UIP and normal lung, with no expression in fibroblastic foci (Fig. 3). Expression of *ALCAM* (mean fold change 0.58, 95% CI 0.40 – 0.85, *P* =0.03) and *FOXP4* (mean fold change 0.70, 95% CI 0.50 – 0.98, *P* = 0.046) were decreased in IPF lung compared to controls (Fig. 2). There was a non-significant trend toward lower *CDKN2B* expression in IPF lung compared to normal lung (mean fold change 0.56, 95% CI 0.33 – 0.94, *P* = 0.07).

## Discussion

In this multi-ethnic GWAS of ~8,000 individuals, we identified associations between HAA phenotypes and the 9p21 region and two other loci also near other RNA genes.

In race/ethnic-specific analyses, we identified a number of genes related to obesity (*GNPDA2*, *ZNF664-FAM101A*, *PFKP*, *SAMD4A*), glycosylation (*GYPC*, *FUT10*), and carbohydrate metabolism (*GNPDA2*, *PFKP*, *SLC45A*). We also identified novel associations with genes that code for a transcription factor (*FOXP4*) that validated in clinical ILD cases, a cell adhesion molecule (*ALCAM*) expressed in the pulmonary microvascular endothelium, a protein involved in reorganization of the actin cytoskeleton (*DAAM1*) that may play a role in pulmonary vascular remodeling, and a (*STK38*) MAP kinase inhibiting protein that protects against acute lung injury.

Our major finding is that *ANRIL* expression is higher in IPF lung compared to normal lung (Additional file 1: Table S14). This discovery came as a result of follow-up on our GWAS finding that HAA, a putative measure of subclinical ILD, is associated with genetic variation at rs7852363, a SNP that sits within the long non-coding RNA *FLJ35282* downstream of the *CDKN2A*/*CDKN2B*/*ANRIL* locus. The latter has been strongly linked to cardiovascular disease [13], diabetes [15], and cancer [16]. Evidence suggests that much of the phenotypic variation linked to 9p21 may be explained by genotypic variation in *ANRIL* [17], a long non-coding RNA (lncRNA) gene that might promote *cis*-acting epigenetic silencing of the 9p21 region by promoting recruitment of polycomb repressive complexes [18], leading to decreased expression of *CDKN2A* and *CDKN2B*. A recent study found that *CDKN2B* is highly methylated in IPF fibroblasts, possibly contributing to increased cyclin-dependent kinase activity and fibroblast proliferation in IPF [19].

Our findings of increased *ANRIL* expression and reduced *CDKN2B* expression in IPF lung support a possible role for disinhibition of cyclin-dependent kinases in IPF progression. The localization of *ANRIL* expression to the airway epithelium, however, suggests that if *ANRIL* plays a role in IPF pathogenesis, it might contribute to an abnormal small airway epithelial response to injury rather than excess fibroblast proliferation. We did not seek validation of rs7852363 in the Framingham Heart Study, since the phenotype demonstrating an association with this locus (basilar HAA peel-to-core ratio) was not available. Although we were unable to demonstrate an association between rs7852363 and clinical ILD case status, the small number of clinical cases, combined with our above findings, suggest that additional work in this area is needed.

We found that the *FOXP4* minor allele was present in 4.8% of ILD cases compared to 2.1% of controls, and *FOXP4* also demonstrated reduced expression in lung tissue from IPF cases compared to controls. *FOXP4* encodes a transcription factor (forkhead box P4) expressed in proximal and distal airway epithelium as well as in subepithelial mesenchyme during lung development [20]. *FOXP4* is also expressed in CD4+ and CD8+ T cells and appears to contribute to cytokine production and memory T cell responses [21], and a recent study reported *FOXP4* as an important regulator of non-small cell lung cancer cells [22]. The specific role of this locus in disease pathogenesis requires further elucidation.

In our replication analysis for percent HAA, only one SNP near the *GNPDA2* demonstrated nominal evidence of association in European ancestry participants from FHS. Given the number of SNPs considered in the replication analysis, nominally significant evidence of association does not pass correction for multiple comparisons, but perhaps offers a suggestion of association. *GNPDA2* catalyzes conversion of D-glucosamine-6-phosphate into D-fructose-6-phosphate, representing a possible relationship with catabolism of N-linked carbohydrates in glycoproteins, similar to *GYPC*, *GFPT1* and *FUT10* also reported in our current GWAS of HAA in MESA. Glycosylation plays a key role in quality control of glycoproteins [23]. In the lung, mucins are heavily glycosylated proteins produced in the epithelium and have been among the most widely replicated association for ILD at *MUC5B* [2, 4, 5]. Altered glycosylation of mucin and/or extracellular matrix proteins in the lung could impair remodeling responses to injury and promote interstitial lung disease [24]. Our novel findings of associations between subclinical ILD and variation at loci responsible for glycosylation suggest that investigation of the role of protein glycosylation in early lung remodeling may provide insights into pathogenesis of ILD.

In analysis of 33 SNPs reported previous GWAS of IPF, we identified association of two *TOLLIP* SNPs with percent HAA traits in MESA African Americans. Combined with the previous association of the same *TOLLIP* SNPs with IPF [3], our identification of TOLLIP SNPs associated with percent HAA suggests these SNPs play a role in subclinical disease progressing toward clinical development of pulmonary fibrosis. *TOLLIP* (also known as Toll interacting protein) has been shown to regulate inflammatory cytokine production in response to interleukin-1 [25], identifying inflammation as a possible mechanism underlying the role of *TOLLIP* in development of IPF.

While we were unable to validate other SNPs that we identified in our GWAS, literature evidence provides some support for a role in ILD pathogenesis for three genes located in or near SNPs identified by our GWAS. Activated leukocyte cell adhesion molecule (CD166; *ALCAM*) is an immunoglobulin-family receptor expressed on activated leukocytes [26] and in some cancers. We found that *ALCAM* is under-expressed in IPF lung. A recent exome sequence analysis of severe early onset COPD exhibited an increased burden of rare deleterious *ALCAM* variants in cases versus controls [27]. *ALCAM* may play a signaling role in extracellular matrix remodeling [28] and is expressed in the pulmonary microvascular endothelium [29]. *DAAM1* encodes for disheveled association activator of morphogenesis 1, a protein involved in actin assembly [30] and endothelial cell proliferation [31]. *DAAM1* is overexpressed in pulmonary arteries in idiopathic pulmonary arterial hypertension [32]. *STK38* encodes for serine-threonine kinase 38, an inhibitor of mitogen-activated protein (MAP) kinase 1 signaling [33] has been linked to protection from nickel-induced acute lung injury [34].

Limitations of the study include a modest sample size for GWAS, particularly among Chinese, such that reported associations should be interpreted with caution. However, the present study is the first and largest GWAS of subclinical ILD phenotypes to date. In addition, data available for replication was limited to European ancestry participants from the Framingham Heart Study who were assessed for percent HAA and basilar percent HAA, but not for basilar peel-core ratio. Accordingly, we could not meaningfully pursue replication of genetic loci identified in non-European ancestry groups from MESA. Further, while the MESA participants were genotyped for GWAS at ~900,000 SNPs using the Affymetrix 6.0 array which was designed to capture common variation across Europeans, East Asians and West Africans [35], we expect that we would have had limited coverage of common variants with MAF less than 0.1 even after incorporating imputation to the 1000 Genomes reference panel. Therefore, despite the fact that the genetic loci reported from our GWAS were subject to strict and systematic filters on imputation quality and minor allele counts, we recognize that many of our reported loci reflect infrequent variants that should be viewed with caution, particularly in the absence of replication and lack of consistent effects across race/ethnic groups. Given that we have focused on a population-based cohort, our study may have reduced power to detect infrequent risk variants seen predominantly in ILD cases. We expect our use of quantitative measures to define subclinical ILD provided us with greater power to identify infrequent variants that confer protection against ILD. Taken together, the limitations of our study underscore the fact that the SNPs and genes implicated by our current work will require additional confirmation through replication and validation in larger sample sizes.

## Conclusions

In summary, we report the first genome-wide association study to probe the genetics of HAA in a general population sample, and the novel finding that *ANRIL*, a long non-coding RNA that promotes loss of cell cycle regulation, is overexpressed in IPF lung. We also report a novel *FOXP4* region SNP that was validated in genetic analysis of ILD cases versus controls, and further demonstrate reduced expression of *FOXP4* in lung tissue from IPF cases compared to controls. Our results suggest morphologic assessment of the lung on CT can build substantially on our knowledge of ILD pathobiology and that disinhibition of cyclin-dependent kinases and abnormalities in protein glycosylation may contribute to ILD risk.

## Additional file

Additional file 1: Online Data Supplement for “Genome-wide association study of subclinical interstitial lung disease in MESA”. (DOCX 14799 kb)

## Abbreviations

CT: Computed tomography
GWAS: Genome-wide association study
HAA: High attenuation area
HU: Hounsfield unit
ILD: Interstitial lung disease
IPF: Idiopathic pulmonary fibrosis
MESA: The Multi-Ethnic Study of Atherosclerosis
SNP: Single nucleotide polymorphism
