# Supplementary material for: Genome-wide association study of subclinical interstitial lung disease in MESA
Source: Respir Res. 2017 May 18;18:97. doi: 10.1186/s12931-017-0581-2 (PMC5437638; doi:10.1186/s12931-017-0581-2)
Supplement: Additional file 1: — Online Data Supplement for “Genome-wide association study of subclinical interstitial lung disease in MESA”. (DOCX 14799 kb) [file 12931_2017_581_MOESM1_ESM.docx]

**Online Data Supplement for “Genome-wide association study of subclinical interstitial lung disease in MESA”**

Ani Manichaikul, Xin-Qun Wang, Li Sun, Josée Dupuis, Alain C. Borczuk, Jennifer N. Nguyen, Ganesh Raghu, Eric A. Hoffman, Suna Onengut-Gumuscu, Emily A. Farber, Joel D. Kaufman, Dan Rabinowitz, Karen D. Hinckley Stukovsky, Steven M. Kawut, Gary M. Hunninghake, George R. Washko, George T. O’Connor, Stephen S. Rich, R. Graham Barr, David J. Lederer

**Study Design**

MESA is a longitudinal study of subclinical cardiovascular disease and risk factors that predict progression to clinically overt cardiovascular disease or progression of the subclinical disease [1]. Between 2000 and 2002, MESA recruited 6,814 men and women 45 to 84 years of age from Forsyth County, North Carolina; New York City; Baltimore; St. Paul, Minnesota; Chicago; and Los Angeles. Exclusion criteria were clinical cardiovascular disease, weight exceeding 136 kg (300 lb.), pregnancy, and impediment to long-term participation.

The MESA Family Study recruited 1,595 African American and Hispanic participants, generally siblings of MESA participants, using the same inclusion and exclusion criteria as MESA except that clinical cardiovascular disease was permitted.

The MESA Air Pollution Study recruited an additional 257 participants from Los Angeles and Riverside County, CA, and Rockland County, NY, using the same criteria as MESA, except that participants were ages 50 to 89 who lived in the area ≥ 50% of the year and had no plans to move in the next five years [2].

**CT measures of percent high attenuation area in MESA**

Lung structure was assessed on the lung fields of cardiac CT scans, which included the entire lung volume from the level of the carina through the lung bases, comprising an estimated 70% of total lung volume. Gated cardiac CT scans were obtained at full inspiration on multidetector-row and electron-beam CT scanners according to a standardized protocol [3]. Two scans were obtained for each participant within the same session.

All analyses were performed at a single reading center by trained readers without knowledge of participant information. Image attenuation was assessed using a modified version of the Pulmonary Analysis Software Suite [4-7]. The scan with the greater volume of lung air was used for analyses, except in cases of discordant scan quality, when the higher-quality scan was analyzed [8]. As air outside the body has a mean attenuation of -1,000 HU, the attenuation of each pixel in the lung regions was corrected to have the value equal to measured pixel attenuation x (-1,000/mean air attenuation).

We defined high attenuation area (percent HAA) as the percentage of lung voxels having a CT attenuation value between -600 and -250 HU, as described previously [9]. Percent HAA was quantified for the caudal 1/3^rd^ of the imaged lung (henceforth termed “basilar percent HAA”). In addition, the basilar peel-core ratio of HAA (henceforth termed “basilar peel-core ratio”) was computed as the percent HAA in the peel region (outer 20mm) of the lung divided by that in the core region for the caudal 1/3^rd^ of the imaged lung. The intraclass correlation coefficient of percent HAA among 100% replicate CT scans was 0.93 (n = 2,653), and the Spearman correlation coefficient of percent HAA between cardiac and full-lung scans (n=42) was 0.87 with a mean difference of 0.2% [9].

**Additional phenotyping of MESA participants**

Pack-years of cigarette smoking were calculated as age of starting to quitting (or current age if current cigarette smoker) × (cigarettes per day/20) using standardized questionnaire items [10]. Ever-smoking was defined as greater than 100 lifetime cigarettes smoked and current smoking as self-report of a cigarette in the last 30 days. Current smoking status was confirmed by cotinine levels in the subset of the cohort with spirometry measures; self-report was generally accurate [11]. Asthma was defined as self-report of physician-diagnosed asthma. Height was measured to the nearest 0.1 cm with the subject in stocking feet and weight was measured to the nearest pound with the subject in light clothing using a balanced scale. Spirometry was conducted in 2004-06 in accordance with the American Thoracic Society/European Respiratory Society guidelines [12] on a dry-rolling-sealed spirometer with automated quality checks (Occupational Marketing, Inc., Houston, TX), as previously described [13], for 3,975 participants.

**Genotype Data**

Participants in the original MESA cohort, the MESA Family Study and the MESA Air Pollution Study who consented to genetic analyses were genotyped in 2009 using the Affymetrix Human SNP array 6.0. Genotype quality control for these data included filter on SNP level call rate < 95%, individual level call rate < 95%, heterozygosity > 53%, described previously [14]. The cleaned genotypic data was deposited with MESA phenotypic data into dbGaP as the MESA SHARe project (study accession phs000209); 8,224 consenting individuals (2,685 White, 2,588 non-Hispanic African-American, 2,174 Hispanic, 777 Chinese) were included, with 897,981 SNPs passing study specific quality control (QC). For GWAS, IMPUTE version 2.2.2 was used to perform imputation for the MESA SHARe participants using the cosmopolitan 1,000 Genomes Phase 1 v3 March 2012 reference set.

**Principal component analysis**

We performed principal component analysis to adjust for population structure among MESA participants, as described previously [14]. We constructed subsets of typed SNPs, thinned for linkage disequilibrium (LD) within each race/ethnic group. We performed Principal Component Analysis (PCA) as implemented in the program SMARTPCA [15, 16] for unrelated subsets of individuals, removing inferred first degree relatives from the analysis. We constructed histograms and QQ-plots to assess symmetry and normality of the distribution of loadings for each of the resulting PCs to determine the optimal number of PCs for genetic association analysis.

**Selection of SNPs for genetic association analysis**

We filtered genotyped SNPs for Hardy-Weinberg Equilibrium (HWE) *P*-value ≥ 10^-5^ and the expected heterozygote count (NHet) > 30 for the SNP, where NHet was quantified as 2 * N * MAF * (1-MAF). We filtered imputed SNPs based on imputation quality > 0.5, using the observed versus expected variance (oevar) quality metric [17] and effective NHet > 30, where effective NHet was computed as NHet x oevar. After applying these filters, 8,736,417; 15,771,936; 11,077,907; and 6,339,878 SNPs remained for genetic association analysis in MESA Whites, African Americans, Hispanics and Chinese, respectively. Quality control metrics for all SNPs reported as genome-wide significant in Table 2 are displayed in Table S15.

**Genetic association analysis**

Within the MESA Lung Fibrosis study, we stratified by race/ethnic group and excluded those individuals with top principal components (PCs) of ancestry > 3.5 SD from the mean within any race/ethnic group. Based on our examination of principal components within each race/ethnic group, we used 3 PCs for analysis of Whites, 1 PC for African Americans, 3 PCs for Hispanics, and 1 PC for Chinese. We Winsorized all phenotypic values more than 3.5 SD from the mean, with the mean and SD calculated separately for each race/ethnic group. Percent HAA, basilar percent HAA, and basilar peel-core ratio were all analyzed on the log-scale.

For analysis of Whites and Chinese, to account for the rare instances in which multiple related individuals were recruited into the cohort, an unrelated subset of individuals was selected using KING [18] to identify at most one individual per pedigree, and we performed linear association analysis of quantitative phenotypes using a likelihood score test with an additive genetic model, using a score test accounting for imputed genotype probabilities as implemented in SNPTEST v2.2.0 [19]. For analysis of African American and Hispanic cohorts (in which substantial family data were available), we performed analysis using an additive 1 d.f. model for genotype dosage with linear mixed-effects as implemented in the package R/GWAF [20].

In analyses of HAA on CT scan, we included covariate adjustment for age, sex, study site, principal components of ancestry, CT scanner, tube current, breath artifacts, height, weight, cigarettes per day (for current smokers only) and pack-years. Within each race/ethnic group, we restricted the dataset to individuals from study sites / CT model pairs for which data were available for at least 20 individuals in the full analyses, or at least 10 individuals in subset analyses (stratified by smoking exposure or other strata). We followed the same procedure for trimming the data set with respect to tube current.

Following stratified analyses within each race/ethnic group, we performed meta-analysis to combine results across all four MESA race/ethnic groups. We used fixed effects meta-analysis to combine estimated effects and standard errors from stratified analyses, as implemented in METAL [21].

We examined genomic control values of all genome-wide association results to assess evidence of residual population stratification, undetected family structure, or other sources of inflation in type I error. The genomic control values of all race/ethnic-specific GWAS ranged from 0.99 – 1.02. In meta-analysis across all ethnic groups, we observed genomic control values of 1.0 – 1.02.

**Replication in the Framingham Heart Study**

We sought replication of SNPs identified at genome-wide significance from the GWAS of HAA in MESA using an independent cohort of individuals from the Framingham Heart Study (FHS). The Framingham Heart Study (FHS) was initiated in 1948 and comprised 5,209 men and women from the Framingham area who were between the ages of 28 and 62 years. Beginning in 1971, FHS enrolled 5,124 additional men and women, who were either offspring of the original cohort or spouses of those offspring. In 2002, a total of 4,095 third generation participants (men and women) were recruited.

During each clinic exam cycle, the participants underwent a detailed examination including physical examination, medical history, laboratory testing, and electrocardiogram. Over the years, other tests (not necessarily performed at every exam cycle) have included pulmonary function, lifestyle, physical function, cognitive function questionnaires, and various noninvasive cardiovascular tests including echocardiograms. Between 2002 and 2005, a subset of the Offspring and Generation 3 cohorts underwent cardiac gated CT scanning of the chest with the same protocol utilized in the National Heart, Lung, and Blood Institute funded MESA [1, 8] cohort. Briefly, the scans were acquired at full inflation using a GE Lightspeed 8-slice scanner (120 KVp, gantry speed 0.5 sec, exposure times 0.33 sec, and a weight adjusted tube current: weight<220 lbs 106 mAs, weight > 220lbs 133 mAs) with a minimum of 10.5cm of data in the z direction (head to foot). The images were reconstructed with a 35cm field-of-view centered on the heart using a 2.5mm slice thickness and the standard reconstruction kernel. A total of 3,010 members of the Offspring (n = 1,193) and Generation 3 (n = 1,817) cohorts had good quality genotypes available from the SNP Health Association Resource (SHARe) project and CT image data that was satisfactory for lung quantitative image analysis.

From a total of 549,781 genotyped SNPs (Affymetrix 500K and MIPS 50K), 412,053 SNPs were used as in the imputation after filtering out SNPs out of HWE (p<1E-6), SNPs with low call rate (missingness >0.03), SNPs with >1000 Mendelian errors, and SNPs with low MAF (<0.01). The software MACH and minimac were used to impute all autosomal and X chromosome SNPs using the November 2010 release of 1000G multi-ethnic panel based on all 1,092 individuals.

**Replication analysis**

FHS represents White participants for whom only percent HAA and basilar percent HAA are available for genetic analysis. Therefore, replication in FHS was focused on 7 SNPs identified in race/ethnic specific GWAS of MESA Whites for percent HAA and basilar percent HAA, as shown in Table 2.

Genetic analyses were performed using linear regression (linear mixed effect models in FHS to account for familial correlation) under an additive model of inheritance. The FHS genetic analyses were adjusted for age, sex, generation (offspring/Generation 3 cohorts), principal components of ancestry, height, weight, cigarettes per day (for current smokers only) and pack-years.

**Validation with ILD cases**

We prospectively enrolled adults with ILD presenting to the interstitial lung disease and lung transplant programs at Columbia University Medical Center between 2007 and 2011. Plasma and buffy coat DNA were obtained at the time of a baseline study visit and stored at -80^o^C until analysis. There were 364 participants (representing Whites, African Americans, Hispanics and Asians) who had DNA available. DNA was genotyped using the Axiom Biobank chip.

*Genotyping and Imputation:* Genotypes for 364 participants completed on the Affymetrix Axiom Biobank chip were called according to the APT Best practices workflow as recommended by the manufacturer (Affymetrix, Santa Clara, CA). SNPs were filtered on call rate > 95%, and the SNP with lower call rate was removed for all pairs of duplicates based on RSID. We further applied a filter of HWE *P*-value > 1E-5 as calculated for samples pooled across race/ethnic groups, as well as in the stratified sample of Whites. A total of 553,114 SNPs remained after QC, including rs2070600. Samples were filtered for call rate > 97%, heterozygosity > 18% and sex mismatch. These high-throughput genotypes were used to carry out relationship inference in KING (1), and one individual was removed from each pair of first degree relatives to construct a subset of unrelated individuals. After applying these individual level filters for genotype QC, we retained data for 349 participants. IMPUTE version 2.2.2 was used to perform imputation of selected genomic regions for the ILD cases using the cosmopolitan 1,000 Genomes Phase 1 v3 March 2012 reference set.

*Matching with MESA Controls:* Before matching the ILD cases with controls the eligible MESA controls, we further used KING (1) to verify there were no unexpected first degree relatives across these two sets of samples. To limit the presence of ILD among the selected controls, we restricted selection of controls to those MESA participants free of self-reported chronic lung diseases other than asthma. PCs of ancestry were computed within race/ethnic groups using up to 53,243 SNPs common to the Affymetrix Axiom Biobank and the Affymetrix 6.0 SNP arrays used to genotype ILD cases and MESA participants, respectively, and those participants identified as outliers in PCs analysis were excluded. Each ILD case was then matched to three eligible MESA controls of the same race/ethnicity. After restricting the ILD cases to those with self-reported White, African American, Hispanic or Asian race/ethnicity for concordance with MESA (6 participants removed), pruning an additional 27 cases identified as outliers in race/ethnic-specific principal components of ancestry, there were 316 ILD cases remaining for genetic analyses.

*Genetic association analysis of ILD cases and matched MESA controls:* We selected SNPs for analysis among the ILD cases based on the results of the primary GWAS of HAA phenotypes in the MESA participants. In race/ethnic specific analysis of Whites, we examined 13 SNPs identified in the GWAS of percent HAA, basilar percent HAA, or basilar peel-core ratio for MESA Whites or in meta-analysis across race/ethnic groups. In combined analysis of ILD cases vs. MESA controls across race/ethnic groups, we examined 16 SNPs presented for the GWAS of HAA traits identified in Whites, African Americans or in combined meta-analysis from Table 2. Analysis of ILD cases vs. race/ethnicity-matched MESA controls was performed by logistic regression with adjustment for sex and race/ethnic-specific PCs among Whites, representing the largest group of cases in the Columbia ILD study. We further performed pooled analysis of ILD cases and controls across all race/ethnic groups using logistic regression with adjustment for sex and PCs of ancestry computed for the pooled set of cases and controls across race/ethnic groups.

**Gene expression analysis**

We measured mRNA expression of four of the genes we identified (*ALCAM*, *FOXP4*, *CDKN2B*, and *ANRIL*, along with the reference gene *GAPDH*) in OCT-embedded fresh frozen lung tissue obtained from 15 adults with IPF and a histologic usual interstitial pneumonia pattern (UIP) and 15 adults without lung disease stored in the Columbia University Pathology Tissue Bank. We chose ANRIL and CDKN2B because of the plausibility that they could be involved in fibroblast proliferation and because it was one of the SNPs that reached genome-wide significance in the meta-analysis across race/ethnic groups. We chose FOXP4 as a representative example of a SNP association with percent HAA and because it validated in clinical ILD cases. ALCAM was chosen as a representative example of a SNP associated with basilar HAA.

RNA was isolated using the RNeasy Plus Mini Kit (Qiagen) and reverse transcribed to cDNA using the High Capacity RNA to cDNA Kit (LifeTechnologies). Quantitative PCR was performed using the StepOnePlus real-time PCR system (LifeTechnologies) in triplicate for 40 cycles for *ALCAM* and *FOXP4* and for 50 cycles for *CDKN2B* and *ANRIL*. ΔCt values were calculated as the difference between the Ct of the transcript of interest and the Ct of *GAPDH* mRNA. Expression of each transcript of interest for each case and control was normalized to the mean Ct value among controls by calculating the “fold difference” for each case and control as 2^-(ΔCt-cmCt)^, where ΔCt is the ΔCt for the transcript of interest and cmCt is the control group mean Ct value for the transcript of interest. “Mean fold changes” in gene expression were calculated using the ΔΔCT method (2^-ΔΔCt^). Standard errors for the mean difference in ΔCt between cases and controls were used to calculate 95% confidence intervals (CIs) for mean fold changes: 2^-[ΔΔCt±(1.96xSE)]^. Wilcoxon rank sum tests were used to compare ΔCt values between cases and controls. The coefficients of variation of the raw Ct values ranged from 0.02% to 1.98%. We also performed *in situ* hybridization to visualize ANRIL transcripts in UIP and control lung tissue (RNAscope® 2.0 High Definition (HD), Advanced Cell Diagnostics, Hayward, CA).

**Table S1:** Detailed race/ethnic-specific results for basilar peel-core ratio for SNPs reported at genome-wide significance in Table 2.

| **SNP ID (Effect / other allele) *Nearest gene(s)*** | **Group** | **Effect allele freq.** | **N** | **Beta** | **SE** | **P-value** |
| --- | --- | --- | --- | --- | --- | --- |
| rs76164182 (C/T)  *SLC45A1* | White | 0.018 | **2314** | **0.210** | **0.038** | **3.2E-08** |
|  | African American | - | - | - | - | - |
|  | Hispanic | - | - | - | - | - |
|  | Chinese | - | - | - | - | - |
|  | Meta-analysis |  |  | 0.210 | 0.038 | 3.2E-08 |
| rs114801796 (C/G)  *FAM69A* | White | 0.017 | **2314** | **0.221** | **0.040** | **4.4E-08** |
|  | African American | - | - | - | - | - |
|  | Hispanic | 0.015 | 1889 | -0.007 | 0.036 | 0.853 |
|  | Chinese | - | - | - | - | - |
|  | Meta-analysis |  |  | 0.094 | 0.027 | 0.001 |
| rs190432524 (T/C)  *GFPT2* | White | 0.009 | **2314** | **0.318** | **0.048** | **5.4E-11** |
|  | African American | 0.027 | 2242 | 0.018 | 0.026 | 0.485 |
|  | Hispanic | 0.058 | 1889 | 0.020 | 0.017 | 0.247 |
|  | Chinese | 0.155 | 664 | 0.007 | 0.016 | 0.650 |
|  | Meta-analysis |  |  | 0.028 | 0.010 | 0.007 |
| rs182717611 (T/C)  *SEH1L* | White | 0.020 | **2314** | **0.170** | **0.031** | **4.7E-08** |
|  | African American | - | - | - | - | - |
|  | Hispanic | 0.015 | 1889 | 0.002 | 0.036 | 0.960 |
|  | Chinese | - | - | - | - | - |
|  | Meta-analysis |  |  | 0.098 | 0.024 | 3.0E-05 |
| rs150377334  (A/C)  *NCOA2* | White | - | - | - | - | - |
|  | African American | 0.009 | **2242** | **0.238** | **0.039** | **7.6E-10** |
|  | Hispanic | - | - | - | - | - |
|  | Chinese | - | - | - | - | - |
|  | Meta-analysis |  |  | 0.238 | **0.039** | **7.6E-10** |
| rs74315875  (G/C)  *SLC25A21* | White | 0.016 | 2314 | 0.009 | 0.026 | 0.731 |
|  | African American | 0.033 | **2242** | **0.116** | **0.019** | **2.5E-09** |
|  | Hispanic | 0.016 | 1889 | -0.043 | 0.038 | 0.254 |
|  | Chinese | - | - | - | - | - |
|  | Meta-analysis |  |  | 0.060 | 0.015 | 3.0E-05 |
| rs7852363 (T/C)  *FLJ35282* | White | 0.132 | 2314 | 0.017 | 0.009 | 0.064 |
|  | African American | 0.379 | 2242 | 0.028 | 0.007 | 5.1E-05 |
|  | Hispanic | 0.162 | 1889 | 0.043 | 0.010 | 1.1E-05 |
|  | Chinese | 0.036 | 664 | 0.019 | 0.029 | 0.520 |
|  | Meta-analysis |  |  | **0.028** | **0.005** | **2.1E-09** |
| rs140142658  (G/A)  *SNAI3-AS1* | White | 0.016 | 2314 | 0.102 | 0.035 | 0.004 |
|  | African American | - | - | **-** | **-** | **-** |
|  | Hispanic | 0.019 | 1889 | 0.158 | 0.031 | 3.7E-07 |
|  | Chinese | - | - | - | - | - |
|  | Meta-analysis |  |  | **0.133** | **0.023** | **9.6E-09** |
| rs3079677  (T/TG)  *D21S2088E* | White | 0.430 | 2314 | 0.025 | 0.007 | 0.001 |
|  | African American | 0.311 | 2242 | 0.019 | 0.009 | 0.026 |
|  | Hispanic | 0.446 | 1889 | 0.021 | 0.008 | 0.008 |
|  | Chinese | 0.578 | 664 | 0.033 | 0.012 | 0.005 |
|  | Meta-analysis |  |  | **0.024** | **0.004** | **2.3E-08** |

Results that reached the Bonferroni threshold for a particular race/ethnic group are shown in **bold**. Results are shown only for subgroups with NHet > 30 (genotyped SNPs) or effective NHet > 30 and imputation quality (observed/expected variance) > 0.5.

**Table S2:** Detailed race/ethnic-specific results for percent HAA for SNPs reported at genome-wide significance in Table 2.

| **SNP ID (Effect / other allele) *Nearest gene(s)*** | **Group** | **Effect allele freq.** | **N** | **Beta** | **SE** | **P-value** |
| --- | --- | --- | --- | --- | --- | --- |
| rs6844387 (A/G)  *GNPDA2* | White | 0.006 | 2434 | 0.634 | 0.088 | **5.5E-13** |
|  | African American | 0.093 | 2470 | 0.006 | 0.016 | 0.696 |
|  | Hispanic | 0.021 | 2065 | 0.018 | 0.041 | 0.655 |
|  | Chinese | - | - | - | - | - |
|  | Meta-analysis |  |  | 0.024 | 0.014 | 0.090 |
| rs2894439 (A/G)  *FOXP4* | White | 0.017 | 2434 | 0.202 | 0.037 | **3.5E-08** |
|  | African American | 0.061 | 2470 | -0.024 | 0.020 | 0.233 |
|  | Hispanic | 0.178 | 2065 | 0.023 | 0.017 | 0.176 |
|  | Chinese | 0.373 | 702 | 0.017 | 0.022 | 0.442 |
|  | Meta-analysis |  |  | 0.024 | 0.011 | 0.027 |
| rs117323377 (G/A) *ZNF664-FAM101A* | White | 0.026 | 2434 | 0.149 | 0.026 | **1.5E-08** |
|  | African American | 0.012 | 2470 | -0.086 | 0.055 | 0.119 |
|  | Hispanic | 0.019 | 2065 | 0.021 | 0.051 | 0.685 |
|  | Chinese | - | - | - | - | - |
|  | Meta-analysis |  |  | 0.090 | 0.022 | 2.7E-05 |
| rs141944608 (T/C)  *DAAM1* | White | 0.009 | 2434 | 0.311 | 0.055 | **1.3E-08** |
|  | African American | - | - | - | - | - |
|  | Hispanic | - | - | - | - | - |
|  | Chinese | - | - | - | - | - |
|  | Meta-analysis |  |  | 0.311 | 0.055 | 1.3E-08 |
| rs79441543 (C/T)  *PFKP* | White | 0.023 | 2434 | -0.015 | 0.039 | 0.693 |
|  | African American | - | - | - | - | - |
|  | Hispanic | 0.097 | 2065 | 0.026 | 0.023 | 0.262 |
|  | Chinese | 0.048 | 702 | 0.615 | 0.097 | **2.8E-10** |
|  | Meta-analysis |  |  | 0.039 | 0.019 | 0.046 |

Results that reached the Bonferroni threshold for a particular race/ethnic group are shown in **bold**. Results are shown only for subgroups with NHet > 30 (genotyped SNPs) or effective NHet > 30 and imputation quality (observed/expected variance) > 0.5.

**Table S3:** Detailed race/ethnic-specific results for basilar percent HAA for SNPs reported at genome-wide significance in Table 2.

| **SNP ID (Effect / other allele) *Nearest gene(s)*** | **Group** | **Effect allele freq.** | **N** | **Beta** | **SE** | **P-value** |
| --- | --- | --- | --- | --- | --- | --- |
| rs138384996 (A/G)  *UBE2U* | White | 0.019 | 2429 | 0.438 | 0.077 | **1.6E-08** |
|  | African American | - | - | - | - | - |
|  | Hispanic | - | - | - | - | - |
|  | Chinese | - | - | - | - | - |
|  | Meta-analysis |  |  | 0.438 | 0.077 | 1.6E-08 |
| rs150536895 (A/G)  *GYPC* | White | 0.009 | 2429 | 0.486 | 0.088 | **3.7E-08** |
|  | African American | - | - | - | - | - |
|  | Hispanic | - | - | - | - | - |
|  | Chinese | - | - | - | - | - |
|  | Meta-analysis |  |  | 0.486 | 0.088 | **3.7E-08** |
| rs146792761 (T/G)  *MIR548A3* | White | 0.012 | 2429 | 0.521 | 0.092 | **1.4E-08** |
|  | African American | - | - | - | - | - |
|  | Hispanic | - | - | - | - | - |
|  | Chinese | - | - | - | - | - |
|  | Meta-analysis |  |  | 0.521 | 0.092 | **1.4E-08** |
| rs145855729 (T/C)  *STK38* | White | - | - | - | - | - |
|  | African American | 0.084 | 2462 | 0.126 | 0.023 | **4.4E-08** |
|  | Hispanic | 0.022 | 2061 | -0.003 | 0.062 | 0.955 |
|  | Chinese | - | - | - | - | - |
|  | Meta-analysis |  |  | 0.110 | 0.022 | 3.3E-07 |
| rs114571830 (A/C)  *FUT10* | White | - | - | - | - | - |
|  | African American | 0.024 | 2462 | 0.227 | 0.041 | **2.6E-08** |
|  | Hispanic | - | - | - | - | - |
|  | Chinese | - | - | - | - | - |
|  | Meta-analysis |  |  | 0.227 | 0.041 | **2.6E-08** |
| rs149416017 (T/G)  *SAMD4A* | White | 0.027 | 2429 | -0.001 | 0.038 | 0.980 |
|  | African American | 0.011 | 2462 | 0.411 | 0.074 | **3.2E-08** |
|  | Hispanic | 0.017 | 2061 | -0.095 | 0.071 | 0.178 |
|  | Chinese | - | - | - | - | - |
|  | Meta-analysis |  |  | 0.051 | 0.031 | 0.094 |
| rs74361312 (G/C)  *SORCS3* | White | - | - | - | - | - |
|  | African American | - | - | - | - | - |
|  | Hispanic | - | - | - | - | - |
|  | Chinese | 0.030 | 701 | 0.495 | 0.090 | **4.0E-08** |
|  | Meta-analysis |  |  | 0.495 | 0.090 | **4.0E-08** |

Results that reached the Bonferroni threshold for a particular race/ethnic group are shown in **bold**. rs6844387 was also highly significantly associated with the basilar HAA, but it showed even stronger association with HAA (see Table 2). Results are shown only for subgroups with NHet > 30 (genotyped SNPs) or effective NHet > 30 and imputation quality (observed/expected variance) > 0.5.

**Table S4:** Summary of genome-wide significant results in the GWAS with additional adjustment for BMI, waist circumference, and diabetes status.

| **Trait** | **Group** | **SNP ID** | **Chr** | **NCBI37 position** | **Nearest gene(s)** | **Effect / other allele** | **Beta** | **SE** | ***P*-value** |
| --- | --- | --- | --- | --- | --- | --- | --- | --- | --- |
| **HAA** | **White** | rs6844387* | 4 | 44,775,446 | *GNPDA2* (upstream) | A/G | 0.568 | 0.084 | 1.1E-11 |
|  |  | rs2894439 | 6 | 41,480,093 | *FOXP4*  (upstream) | A/G | 0.197 | 0.037 | 6.9E-08 |
|  |  | rs117323377 | 12 | 124,715,694 | *ZNF664-FAM101A*  (intronic) | G/A | 0.151 | 0.026 | 9.6E-09 |
|  |  | rs141944608 | 14 | 59,455,322 | *DAAM1* (upstream) | T/C | 0.303 | 0.054 | 2.7E-08 |
|  | **Chinese** | rs79441543 | 10 | 3,132,994 | *PFKP*  (intronic) | C/T | 0.603 | 0.095 | 2.7E-10 |
| **Basilar HAA** | **White** | rs138384996 | 1 | 64,724,706 | *UBE2U*  (downstream) | A/G | 0.451 | 0.078 | 7.2E-09 |
|  |  | rs150536895 | 2 | 127,254,486 | *GYPC*  (upstream) | A/G | 0.484 | 0.088 | 4.3E-08 |
|  |  | rs146792761 | 3 | 103,949,823 | *ALCAM*  (upstream) | T/G | 0.525 | 0.092 | 1.1E-08 |
|  | **African**  **American** | rs145855729 | 6 | 36,495,778 | *STK38*  (intronic) | T/C | 0.125 | 0.023 | 3.8E-08 |
|  |  | rs114571830 | 8 | 32,973,995 | *FUT10*  (downstream) | A/C | 0.218 | 0.040 | 5.9E-08 |
|  |  | rs149416017 | 14 | 55,227,152 | *SAMD4A*  (intronic) | T/G | 0.379 | 0.074 | 3.1E-07 |
|  | **Chinese** | rs74361312 | 10 | 107,354,812 | *SORCS3*  (downstream) | G/C | 0.457 | 0.089 | 2.4E-07 |
| **Basilar HAA peel-to-core ratio** | **White** | rs76164182 | 1 | 8,291,827 | *SLC45A1*  (upstream) | C/T | 0.209 | 0.038 | 3.5E-08 |
|  |  | rs114801796 | 1 | 93,408,101 | *FAM69A*  (intronic) | C/G | 0.220 | 0.040 | 5.3E-08 |
|  |  | rs190432524 | 5 | 179,742,263 | *GFPT2*  (intronic) | T/C | 0.317 | 0.048 | 5.7E-11 |
|  |  | rs182717611 | 18 | 12,955,146 | *SEH1L*  (intronic) | T/C | 0.170 | 0.031 | 5.4E-08 |
|  | **African**  **American** | rs150377334 | 8 | 71,177,444 | *NCOA2*  (intronic) | A/C | 0.238 | 0.038 | 5.59E-10 |
|  |  | rs74315875 | 14 | 37,335,713 | *SLC25A21*  (intronic) | G/C | 0.113 | 0.019 | 6.7E-09 |
| **Basilar HAA peel-to-core ratio** | **Meta-analysis** | rs7852363 | 9 | 22,790,485 | *FLJ35282*  (intronic) | T/C | 0.027 | 0.005 | 8.5E-09 |
|  |  | rs140142658 | 16 | 88,732,573 | *SNAI3-AS1*  (intronic) | G/A | 0.136 | 0.023 | 4.70E-09 |
|  |  | rs3079677 | 21 | 24,735,151 | *D21S2088E*  (intronic) | T/TG | 0.024 | 0.004 | 2.7E-08 |

Results are presented based on the basic model of genetic association, including adjustment for age, sex, study site, CT scanner, principal components of ancestry, height, tube current, BMI, waist circumference, diabetes, breath artifacts, cigarettes per day, and pack-years. *Statistical association for rs6844387 also reached the genome-wide significance threshold for basilar percent HAA, but the association was stronger for percent HAA.

**Table S5:** Summary of genome-wide significant results in the GWAS with additional adjustment for percent emphysema.

| **Trait** | **Group** | **SNP ID** | **Chr** | **NCBI37 position** | **Nearest gene(s)** | **Effect / other allele** | **Beta** | **SE** | ***P*-value** |
| --- | --- | --- | --- | --- | --- | --- | --- | --- | --- |
| **HAA** | **White** | rs6844387* | 4 | 44,775,446 | *GNPDA2* (upstream) | A/G | 0.401 | 0.060 | 2.8E-11 |
|  |  | rs2894439 | 6 | 41,480,093 | *FOXP4*  (upstream) | A/G | 0.077 | 0.026 | 2.5E-03 |
|  |  | rs117323377 | 12 | 124,715,694 | *ZNF664-FAM101A*  (intronic) | G/A | 0.076 | 0.019 | 3.8E-05 |
|  |  | rs141944608 | 14 | 59,455,322 | *DAAM1* (upstream) | T/C | 0.156 | 0.038 | 3.6E-05 |
|  | **Chinese** | rs79441543 | 10 | 3,132,994 | *PFKP*  (intronic) | C/T | 0.501 | 0.065 | 1.6E-14 |
| **Basilar HAA** | **White** | rs138384996 | 1 | 64,724,706 | *UBE2U*  (downstream) | A/G | 0.272 | 0.056 | 1.3E-06 |
|  |  | rs150536895 | 2 | 127,254,486 | *GYPC*  (upstream) | A/G | 0.292 | 0.070 | 3.3E-05 |
|  |  | rs146792761 | 3 | 103,949,823 | *ALCAM*  (upstream) | T/G | 0.379 | 0.072 | 1.4E-07 |
|  | **African**  **American** | rs145855729 | 6 | 36,495,778 | *STK38*  (intronic) | T/C | 0.092 | 0.019 | 1.6E-06 |
|  |  | rs114571830 | 8 | 32,973,995 | *FUT10*  (downstream) | A/C | 0.150 | 0.034 | 9.2E-06 |
|  |  | rs149416017 | 14 | 55,227,152 | *SAMD4A*  (intronic) | T/G | 0.329 | 0.062 | 9.3E-08 |
|  | **Chinese** | rs74361312 | 10 | 107,354,812 | *SORCS3*  (downstream) | G/C | 0.345 | 0.064 | 7.8E-08 |
| **Basilar HAA peel-to-core ratio** | **White** | rs76164182 | 1 | 8,291,827 | *SLC45A1*  (upstream) | C/T | 0.210 | 0.038 | 2.7E-08 |
|  |  | rs114801796 | 1 | 93,408,101 | *FAM69A*  (intronic) | C/G | 0.219 | 0.040 | 5.6E-08 |
|  |  | rs190432524 | 5 | 179,742,263 | *GFPT2*  (intronic) | T/C | 0.322 | 0.049 | 3.1E-11 |
|  |  | rs182717611 | 18 | 12,955,146 | *SEH1L*  (intronic) | T/C | 0.171 | 0.031 | 4.0E-08 |
|  | **African**  **American** | rs150377334 | 8 | 71,177,444 | *NCOA2*  (intronic) | A/C | 0.238 | 0.037 | 3.1E-10 |
|  |  | rs74315875 | 14 | 37,335,713 | *SLC25A21*  (intronic) | G/C | 0.116 | 0.019 | 1.8E-09 |
| **Basilar HAA peel-to-core ratio** | **Meta-analysis** | rs7852363 | 9 | 22,790,485 | *FLJ35282*  (intronic) | T/C | 0.027 | 0.005 | 1.4E-08 |
|  |  | rs140142658 | 16 | 88,732,573 | *SNAI3-AS1*  (intronic) | G/A | 0.134 | 0.023 | 7.7E-09 |
|  |  | rs3079677 | 21 | 24,735,151 | *D21S2088E*  (intronic) | T/TG | 0.023 | 0.004 | 3.1E-08 |

Results are presented based on the basic model of genetic association, including adjustment for age, sex, study site, principal components of ancestry, CT scanner, tube current, breath artifacts, height, weight, cigarettes per day (for current smokers only), pack-years and percent emphysema at -950 HU. *Statistical association for rs6844387 also reached the genome-wide significance threshold for basilar percent HAA, but the association was stronger for percent HAA.

**Table S6:** Analysis of genetic association with percent emphysema for SNPs identified in the GWAS of subclincal ILD traits.

| **Subclinical ILD GWAS**  **Trait** | **Group** | **SNP ID** | **Chr** | **NCBI37 position** | **Nearest gene(s)** | **Effect / other allele** | **Beta** | **SE** | ***P*-value** |
| --- | --- | --- | --- | --- | --- | --- | --- | --- | --- |
| **HAA** | **White** | rs6844387* | 4 | 44,775,446 | *GNPDA2* (upstream) | A/G | -0.221 | 0.114 | 0.052 |
|  |  | rs2894439 | 6 | 41,480,093 | *FOXP4*  (upstream) | A/G | -0.358 | 0.078 | 4.1E-06 |
|  |  | rs117323377 | 12 | 124,715,694 | *ZNF664-FAM101A*  (intronic) | G/A | -0.219 | 0.057 | 1.2E-04 |
|  |  | rs141944608 | 14 | 59,455,322 | *DAAM1* (upstream) | T/C | -0.353 | 0.106 | 8.2E-04 |
|  | **Chinese** | rs79441543 | 10 | 3,132,994 | *PFKP*  (intronic) | C/T | -0.117 | 0.103 | 0.255 |
| **Basilar HAA** | **White** | rs138384996 | 1 | 64,724,706 | *UBE2U*  (downstream) | A/G | -0.161 | 0.087 | 0.065 |
|  |  | rs150536895 | 2 | 127,254,486 | *GYPC*  (upstream) | A/G | -0.288 | 0.103 | 5.0E-03 |
|  |  | rs146792761 | 3 | 103,949,823 | *ALCAM*  (upstream) | T/G | -0.211 | 0.111 | 0.058 |
|  | **African**  **American** | rs145855729 | 6 | 36,495,778 | *STK38*  (intronic) | T/C | -0.091 | 0.033 | 6.4E-03 |
|  |  | rs114571830 | 8 | 32,973,995 | *FUT10*  (downstream) | A/C | -0.208 | 0.059 | 4.1E-04 |
|  |  | rs149416017 | 14 | 55,227,152 | *SAMD4A*  (intronic) | T/G | -0.191 | 0.106 | 0.073 |
|  | **Chinese** | rs74361312 | 10 | 107,354,812 | *SORCS3*  (downstream) | G/C | -0.199 | 0.095 | 0.037 |
| **Basilar HAA peel-to-core ratio** | **White** | rs76164182 | 1 | 8,291,827 | *SLC45A1*  (upstream) | C/T | -0.073 | 0.088 | 0.408 |
|  |  | rs114801796 | 1 | 93,408,101 | *FAM69A*  (intronic) | C/G | 0.053 | 0.091 | 0.560 |
|  |  | rs190432524 | 5 | 179,742,263 | *GFPT2*  (intronic) | T/C | -0.093 | 0.104 | 0.372 |
|  |  | rs182717611 | 18 | 12,955,146 | *SEH1L*  (intronic) | T/C | -0.008 | 0.074 | 0.918 |
|  | **African**  **American** | rs150377334 | 8 | 71,177,444 | *NCOA2*  (intronic) | A/C | -0.058 | 0.098 | 0.555 |
|  |  | rs74315875 | 14 | 37,335,713 | *SLC25A21*  (intronic) | G/C | -0.054 | 0.051 | 0.294 |
| **Basilar HAA peel-to-core ratio** | **Meta-analysis** | rs7852363 | 9 | 22,790,485 | *FLJ35282*  (intronic) | T/C | -0.009 | 0.013 | 0.492 |
|  |  | rs140142658 | 16 | 88,732,573 | *SNAI3-AS1*  (intronic) | G/A | 0.034 | 0.059 | 0.568 |
|  |  | rs3079677 | 21 | 24,735,151 | *D21S2088E*  (intronic) | T/TG | 0.0004 | 0.011 | 0.972 |

Results for genetic association of percent emphysema at -950 HU are presented with covariate adjustment for age, sex, study site, principal components of ancestry, CT scanner, tube current, breath artifacts, height, weight, cigarettes per day (for current smokers only), and pack-years. *Statistical association for rs6844387 also reached the genome-wide significance threshold for basilar percent HAA, but the association was stronger for percent HAA.

**Table S7:** Descriptive statistics for the Framingham Heart Study with comparison to MESA White.

|  | **MESA White** | **Framingham Heart Study** |
| --- | --- | --- |
| **Participant characteristics*** |  |  |
| No. subjects | 2434 | 1894 |
| Women | 1267 (52.1) | 932 (49.2) |
| Age, years | 63 [54, 71] | 54  [48,62] |
| Height, cm | 168.7  [161.7, 176.2] | 169.5  [162.6, 175.9] |
| Weight, lbs | 172.4 [148.0, 198.5] | 178.0  [152.0, 204.0] |
|  |  |  |
| Ever-smoke (yes/no) | 1345 (55.3) | 902 (47.6) |
| Current-smoke (yes/no) | 269 (11.4) | 123 (6.5) |
| Pack-years of smoking† | 18.8 [6.5, 37.5] | 12.6  [5.0, 27.0] |
| Cigarettes per day† | 15 [9, 20] | 15  [8, 20] |
|  |  |  |
| **Measures of HAA** |  |  |
| No. subjects | 2434 | 1894 |
| Percent HAA | 3.9 [3.3, 4.8] | 1.8  [1.5, 2.1] |
| Basilar percent HAA | 4.3 [3.4, 5.7] | 1.9  [1.6, 2.2] |
|  |  |  |
| **Pulmonary function*** |  |  |
| No. subjects | 1354 | 1800 |
| FEV_1_ (mL) | 2515 [2036, 3086] | 3055  [2508, 3720] |
| FVC (mL) | 3444  [2808, 4180] | 4050  [3370, 4960] |
| FEV_1_/FVC | 0.743 [0.690 0.786] | 0.75  [0.71, 0.80] |

Data are presented as n (%) for binary measures or median [IQR] for continuous measure.

*Summary statistics are reported for the subset of individuals with data available for at least one of the HAA phenotypes.

†Pack-years of smoking are reported for ever-smokers only. Cigarettes per day are reported for current smokers only.

**Table S8:** Results of replication in the Framingham Heart Study.

| **Trait** | **SNP ID** | **Nearest gene(s)** | **Effect/Other allele** | **Effect allele freq** | **Imputation quality** | **N** | **Beta** | **SE** | **P-value** | **Effective NHet** |
| --- | --- | --- | --- | --- | --- | --- | --- | --- | --- | --- |
| Percent HAA | rs6844387 | *GNPDA2* | A/G | 0.004 | 0.881 | 1894 | 0.196 | 0.090 | 0.029 | 13.4 |
|  | rs2894439 | *FOXP4* | A/G | 0.022 | 0.556 | 1894 | 0.036 | 0.050 | 0.467 | 47.1 |
|  | rs117323377 | *ZNF664*-*FAM101A* | G/A | 0.022 | 0.764 | 1894 | 0.057 | 0.041 | 0.161 | 58.7 |
|  | rs141944608 | *DAAM1* | T/C | 0.003 | 0.496 | 1894 | --- | --- | --- | 6.7 |
| Basilar percent HAA | rs138384996 | *UBE2U* | A/G | 0.017 | 0.558 | 1894 | -0.018 | 0.060 | 0.763 | 37.5 |
|  | rs150536895 | *GYPC* | A/G | 0.006 | 0.643 | 1894 | 0.037 | 0.110 | 0.736 | 18.5 |
|  | rs146792761 | *ALCAM* | T/G | 0.006 | 0.457 | 1894 | 0.044 | 0.121 | 0.716 | 11.8 |

Regression analysis of basilar HAA with based on an additive model of genetic association with adjustment for age, sex, generation (offspring/Generation 3 cohorts), principal components of ancestry, height, weight, cigarettes per day (for current smokers only) and pack-years. FHS is a single center study and used a single CT scanner, so there was no need to adjust for study site and CT scanner in these analyses. Breath artifacts were not assessed in FHS. We restrict display of results to those SNPs with effective NHet > 10.

**Table S9:** Characteristics of Columbia ILD participants with available genotyping

| **Characteristic** | **Genetic Participants** |
| --- | --- |
| Age, years | 57.3 ± 11.8 |
| Female | 43% |
| Race/ethnicity |  |
| White | 75% |
| African American | 16% |
| Hispanic | 5% |
| Asian | 5% |
| Diagnosis |  |
| Idiopathic Pulmonary Fibrosis | 34% |
| Other ILD | 66% |
| Forced vital capacity, % predicted | 55.4 ± 17.9 |
| Diffusing capacity for carbon monoxide, % predicted | 32.5 ± 11.7 |
| Body mass index, kg/m^2^ | 27.6 ± 5.4 |

Data are mean ± standard deviation and percentage.

MESA controls were matched to Columbia ILD genetic particiipants on race/ethnicity. The MESA controls were 53.2% female. The mean (SD) age in years of MESA controls 60.4 (9.5). The mean (SD) BMI of MESA controls was 28.6 (5.3).

**Table S10:** Results for the analysis of selected SNPs in stratified analysis of Whites comparing ILD cases versus matched MESA controls.

| **RSID** | **Nearest gene(s)** | **Effect/Other allele** | **Effect allele frequency** | | **N** | **N cases** | **Beta** | **SE** | **One-sided P-value** |
| --- | --- | --- | --- | --- | --- | --- | --- | --- | --- |
|  |  |  | **Case** | **Control** |  |  |  |  |  |
| rs6844387 | *GNPDA2* | A/G | 0.002 | 0.010 | 912 | 228 | --- | --- | --- |
| rs2894439 | *FOXP4* | A/G | 0.048 | 0.021 | 912 | 228 | 0.923 | 0.372 | 0.0065 |
| rs117323377 | *ZNF664-FAM101A* | G/A | 0.033 | 0.026 | 912 | 228 | 0.181 | 0.352 | 0.304 |
| rs141944608 | *DAAM1* | T/C | 0.002 | 0.009 | 912 | 228 | --- | --- | --- |
| rs138384996 | *UBE2U* | A/G | 0.024 | 0.019 | 912 | 228 | --- | --- | --- |
| rs150536895 | *GYPC* | A/G | 0.009 | 0.013 | 912 | 228 | --- | --- | --- |
| rs146792761 | *ALCAM* | T/G | 0.004 | 0.010 | 912 | 228 | --- | --- | --- |
| rs76164182 | *SLC45A1* | C/T | 0.026 | 0.017 | 912 | 228 | --- | --- | --- |
| rs114801796 | *FAM69A* | C/G | 0.011 | 0.017 | 912 | 228 | --- | --- | --- |
| rs190432524 | *GFPT2* | T/C | 0.004 | 0.008 | 912 | 228 | --- | --- | --- |
| rs182717611 | *SEH1L* | T/C | 0.019 | 0.019 | 912 | 228 | --- | --- | --- |
| rs7852363 | *FLJ35282* | T/C | 0.105 | 0.129 | 912 | 228 | -0.222 | 0.196 | 0.871 |
| rs140142658 | *SNAI3-AS1* | G/A | 0.022 | 0.013 | 912 | 228 | 0.713 | 0.480 | 0.069 |
| rs3079677 | *D21S2088E* | T/TG | 0.411 | 0.425 | 912 | 228 | -0.176 | 0.133 | 0.907 |

Genetic analyses were performed by logistic regression with adjustment for sex and race/ethnic-specific PCs computed among Whites. Statistical significance is reported at the nominal level for one-sided tests examining concordance with the direction of effect observed for the reported GWAS signals in Table 2. Results are shown only for those SNPs with effective NHet > 5 in both cases and controls.

**Table S11:** SNPs selected for look-up in MESA GWAS results based on previous reports of association with IPF [22-25].

| **RSID** | **Author** |
| --- | --- |
| rs10902081 | Seibold |
| rs10902089 | Seibold |
| rs111521887 | Noth |
| rs11191865 | Fingerlin |
| rs12610495 | Fingerlin |
| rs1278769 | Fingerlin |
| rs12804004 | Seibold |
| rs17690703 | Noth |
| rs1981997 | Fingerlin |
| rs2034650 | Fingerlin |
| rs2076295 | Fingerlin |
| rs2609255 | Fingerlin |
| rs2672794 | Seibold |
| rs2736100 | Fingerlin & Mushiroda |
| rs28403537 | Seibold |
| rs28654232 | Seibold |
| rs34595903 | Seibold |
| rs35288961 | Seibold |
| rs35619543 | Seibold |
| rs35671223 | Seibold |
| rs35705950 | Noth & Seibold |
| rs41453346 | Seibold |
| rs41480348 | Seibold |
| rs4727443 | Fingerlin |
| rs55846509 | Seibold |
| rs5743890 | Noth |
| rs5743894 | Noth |
| rs6793295 | Fingerlin |
| rs7127117 | Seibold |
| rs7144383 | Noth |
| rs7934606 | Fingerlin & Seibold |
| rs868903 | Seibold |
| rs9667239 | Seibold |

**Table S12:** Detailed race/ethnic-specific results for percent HAA for SNPs statistically significant association in at least one race/ethnic group in the look-up of previously reported IPF SNPs.

| **Authors** | **SNP ID (Effect / other allele) *Nearest gene(s)*** | **Group** | **MAF** | **N** | **Beta** | **SE** | **P-value** |
| --- | --- | --- | --- | --- | --- | --- | --- |
| Noth et al. | **rs111521887**  (G/C)  *TOLLIP* (intronic) | White | 0.185 | 2434 | 0.013 | 0.012 | 0.2534 |
|  |  | African American | 0.055 | 2470 | 0.083 | 0.025 | **0.0011** |
|  |  | Hispanic | 0.094 | 2065 | 0.020 | 0.024 | 0.3874 |
|  |  | Chinese | 0.013 | 702 | -0.131 | 0.139 | 0.3454 |
|  |  | Meta-analysis |  |  | 0.024 | 0.010 | 0.0129 |
| Noth et al. | **rs5743894**  (C/T)  *TOLLIP* (intronic) | White | 0.192 | 2434 | 0.015 | 0.011 | 0.1828 |
|  |  | African American | 0.056 | 2470 | 0.082 | 0.025 | **0.0009** |
|  |  | Hispanic | 0.100 | 2065 | 0.017 | 0.022 | 0.4452 |
|  |  | Chinese | 0.014 | 702 | -0.126 | 0.138 | 0.3603 |
|  |  | Meta-analysis |  |  | 0.025 | 0.009 | 0.0087 |

Results that reached the Bonferroni threshold for a particular race/ethnic group are shown in **bold**.

**Table S13:** Detailed race/ethnic-specific results for basilar percent HAA for SNPs statistically significant association in at least one race/ethnic group in the look-up of previously reported IPF SNPs.

| **Authors** | **SNP ID (Effect / other allele) *Nearest gene(s)*** | **Group** | **MAF** | **N** | **Beta** | **SE** | **P-value** |
| --- | --- | --- | --- | --- | --- | --- | --- |
| Noth et al. | **rs111521887**  (G/C)  *TOLLIP* (intronic) | White | 0.185 | 2429 | 0.016 | 0.017 | 0.3412 |
|  |  | African American | 0.055 | 2462 | 0.113 | 0.034 | **0.0008** |
|  |  | Hispanic | 0.094 | 2061 | 0.022 | 0.031 | 0.4766 |
|  |  | Chinese | 0.013 | 701 | -0.205 | 0.191 | 0.2846 |
|  |  | Meta-analysis |  |  | 0.032 | 0.014 | 0.0193 |
| Noth et al. | **rs5743894**  (C/T)  *TOLLIP* (intronic) | White | 0.192 | 2429 | 0.018 | 0.017 | 0.2683 |
|  |  | African American | 0.056 | 2462 | 0.113 | 0.033 | **0.0007** |
|  |  | Hispanic | 0.100 | 2061 | 0.018 | 0.029 | 0.5483 |
|  |  | Chinese | 0.014 | 701 | -0.194 | 0.188 | 0.3040 |
|  |  | Meta-analysis |  |  | 0.032 | 0.013 | 0.0147 |

Results that reached the Bonferroni threshold for a particular race/ethnic group are shown in **bold**.

**Table S14:** Summary of evidence identified by the current manuscript for *ANRIL* and neighboring genes.

| **Approach** | **Study subjects / scope** | **SNP or gene(s) implicated** | **Summary of findings** |
| --- | --- | --- | --- |
| Genetic association analysis | GWAS analysis in MESA | rs7852363 in *FLJ35282* ~670 kb downstream of *ANRIL* and ~781 kb upstream of *CDKN2B* | Minor allele T associated with increased basilar peel-core HAA (*P*=2.1x10^-9^) with consistent direction of effect in all MESA race/ethnic groups |
|  | Validation analysis in Columbia ILD cases vs. matched MESA controls |  | Minor allele T does not demonstrate evidence of association with increased risk of ILD (one-sided *P*-value=0.871) |
| mRNA expression | 15 adults with IPF compared to 15 adults without lung disease from the Columbia University Pathology Tissue Bank (CUPTB) | *ANRIL* | Increased mRNA expression in IPF vs. normal lung (mean fold change 3.1, *P*=0.001) |
|  |  | *CDKN2B* | Trend toward lower mRNA expression in IPF vs. normal lung (mean fold change 0.56, *P*=0.07) |
| In situ hybridization | UIP and normal lung tissue from the CUPTB | *ANRIL* | mRNA localized to airway epithelium in both UIP and normal lung, with no expression in fibroblastic foci |

**Table S15:** Genotyping and imputation quality metrics for SNPs reported as statistically significant in Table 2Ta.

| **Group** | **SNP ID** | **Chr** | **NCBI37 position** | **Type** | **HWE P-value**  **(genotyped only)** | **Imputation quality (observed/expected variance)** | **NHet (genotyped) or effective NHet (imputed)** |
| --- | --- | --- | --- | --- | --- | --- | --- |
| Meta-analysis | rs7852363 | 9 | 22,790,485 | Imputed | --- | 0.981/  0.962/  0.986/  1.019 | 521.35/  1015.01/  506.28/  46.76 |
|  | rs140142658 | 16 | 88,732,573 | Imputed | --- | 0.557/  0.417  0.720/  0.035 | 40.21/  40.53/  31.17/  0.031 |
|  | rs3079677 | 21 | 24,735,151 | Imputed | --- | 0.778/  0.690/  0.779/  0.862 | 881.90/  663.72/  727.31/  279.24 |
| White | rs6844387 | 4 | 44,775,446 | Genotyped | 0.0805 | --- | 30.80 |
|  | rs2894439 | 6 | 41,480,093 | Imputed | --- | 0.745 | 61.41 |
|  | rs117323377 | 12 | 124,715,694 | Imputed | --- | 0.862 | 107.81 |
|  | rs141944608 | 14 | 59455322 | Imputed | --- | 0.761 | 31.57 |
|  | rs138384996 | 1 | 64,724,706 | Imputed | --- | 0.528 | 48.55 |
|  | rs150536895 | 2 | 127,254,486 | Imputed | --- | 0.814 | 33.49 |
|  | rs146792761 | 3 | 103,949,823 | Imputed | --- | 0.554 | 32.29 |
|  | rs76164182 | 1 | 8,291,827 | Imputed | --- | 0.567 | 46.73 |
|  | rs114801796 | 1 | 93,408,101 | Imputed | --- | 0.504 | 39.92 |
|  | rs190432524 | 5 | 179,742,263 | Imputed | --- | 0.720 | 30.14 |
|  | rs182717611 | 18 | 12,955,146 | Imputed | --- | 0.673 | 59.99 |
| Chinese | rs79441543 | 10 | 3,132,994 | Imputed | --- | 0.526 | 33.45 |
|  | rs74361312 | 10 | 107354812 | Imputed | --- | 0.820 | 33.28 |
| African American | rs145855729 | 6 | 36,495,778 | Imputed | --- | 0.898 | 338.37 |
|  | rs114571830 | 8 | 32,973,995 | Imputed | --- | 0.938 | 108.30 |
|  | rs149416017 | 14 | 55,227,152 | Imputed | --- | 0.587 | 32.10 |
|  | rs150377334 | 8 | 71,177,444 | Imputed | --- | 0.771 | 32.49 |
|  | rs74315875 | 14 | 37,335,713 | Imputed | --- | 0.865 | 125.04 |

HWE P-values and imputation quality for meta-analysis are reported for White / African American / Hispanic / Chinese ethnic groups, respectively.

**Figure S1:** Manhattan plots for the GWAS of (A) percent HAA, (B) basilar percent HAA, and (C) basilar peel-core ratio in combined meta-analysis across race/ethnic groups in MESA.

(A)

**
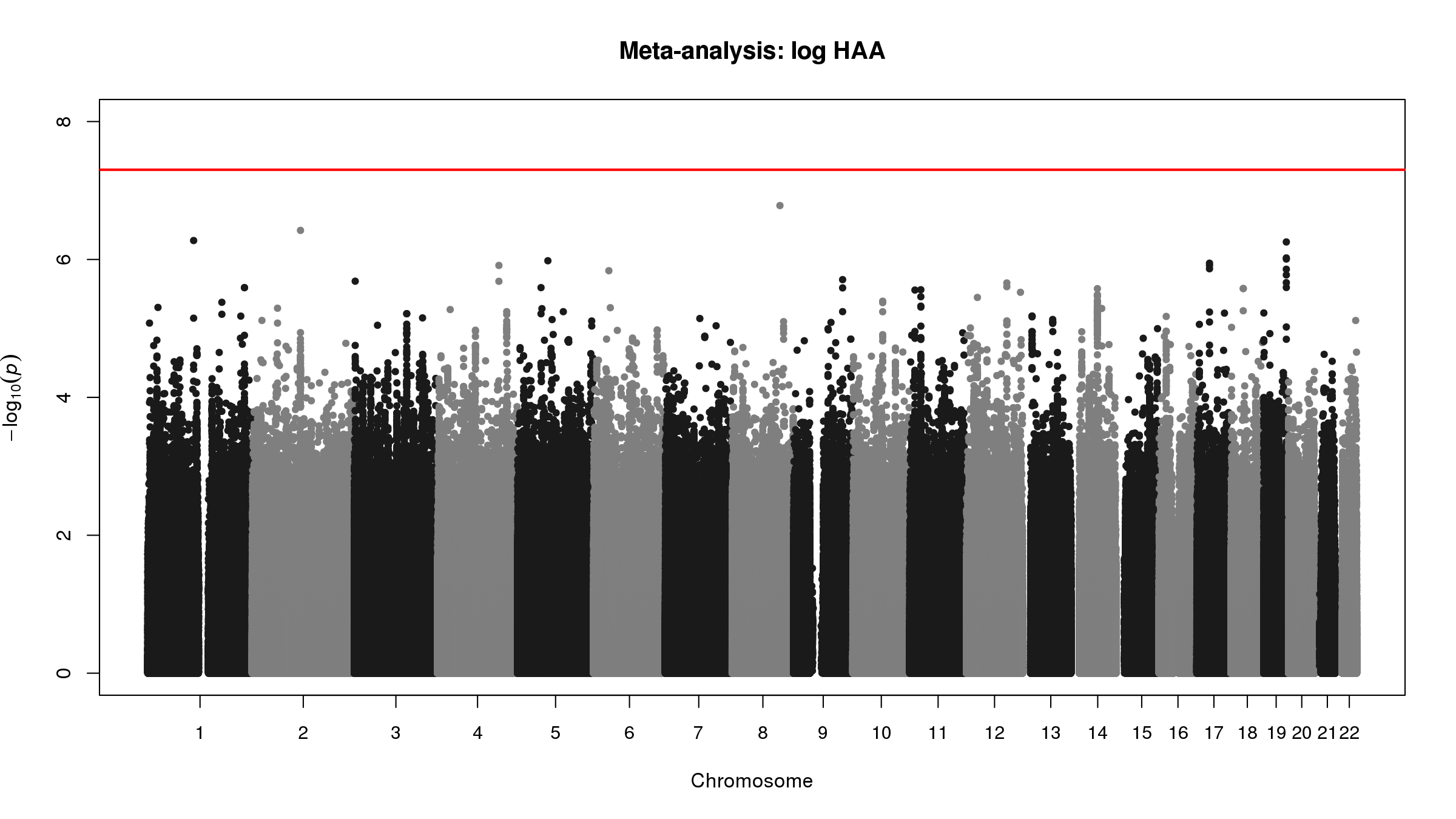
**

(B)

**
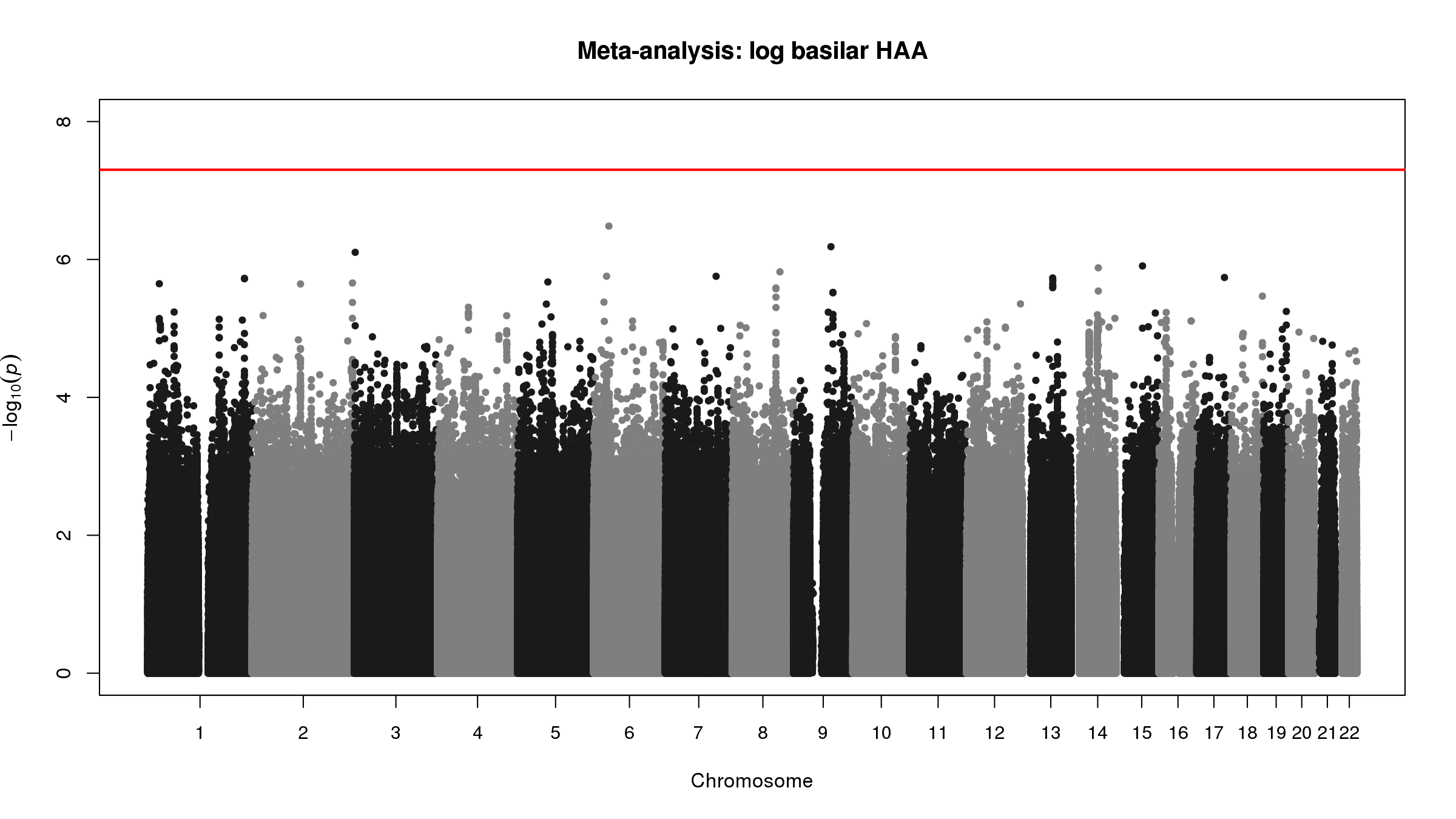
**

(C)

**
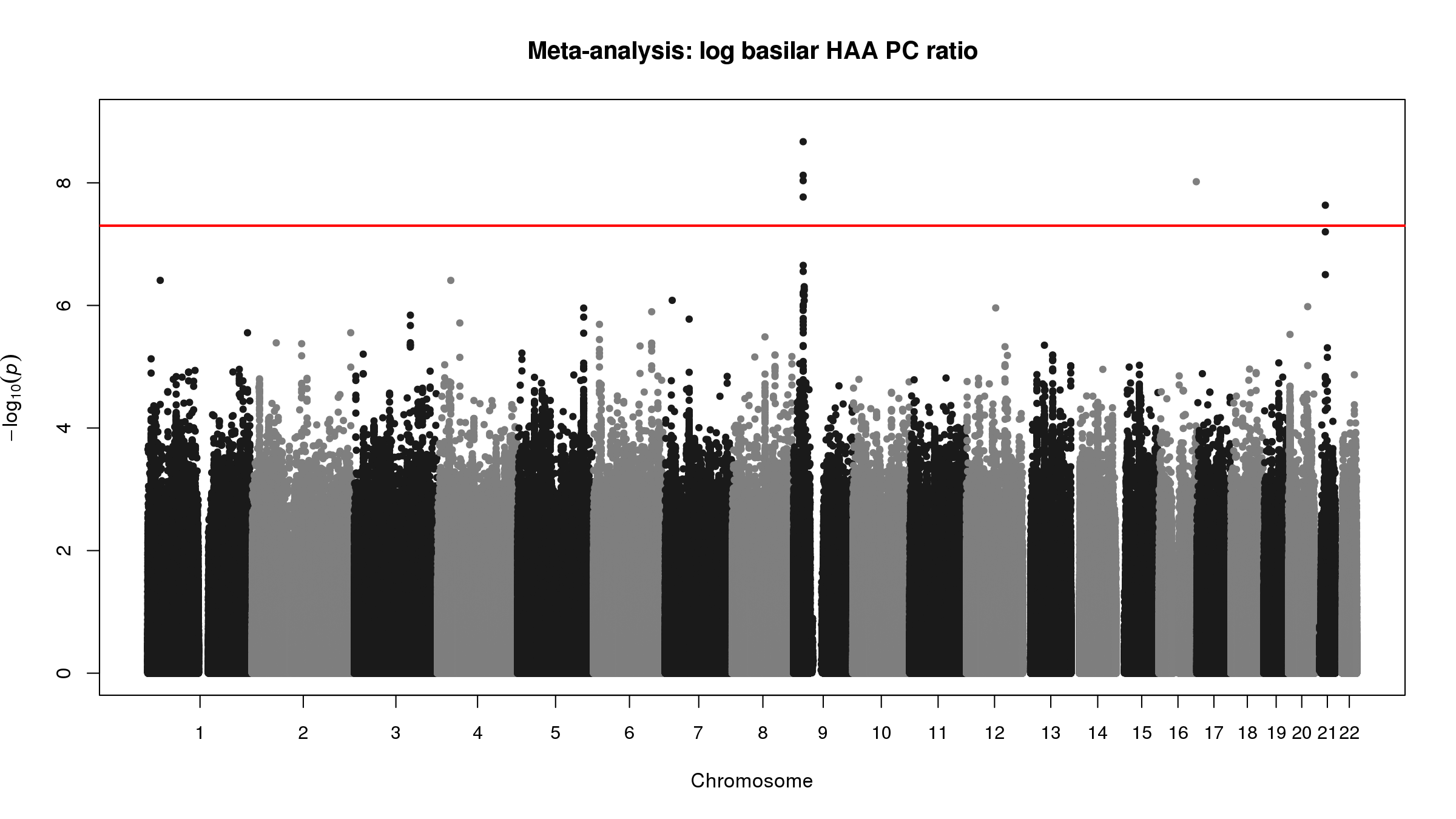
**

**Figure S2:** Quantile-quantile plots for the GWAS of (A) percent HAA, (B) basilar percent HAA, and (C) basilar peel-core ratio in combined meta-analysis across race/ethnic groups in MESA.

(A) (B)


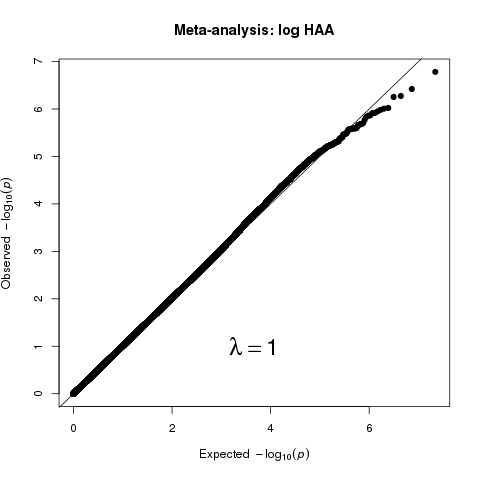

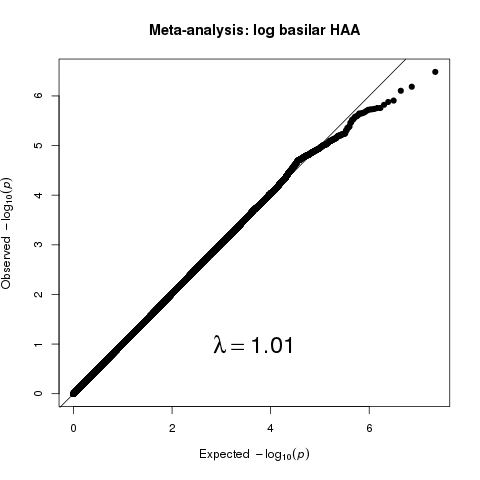


(C)

**
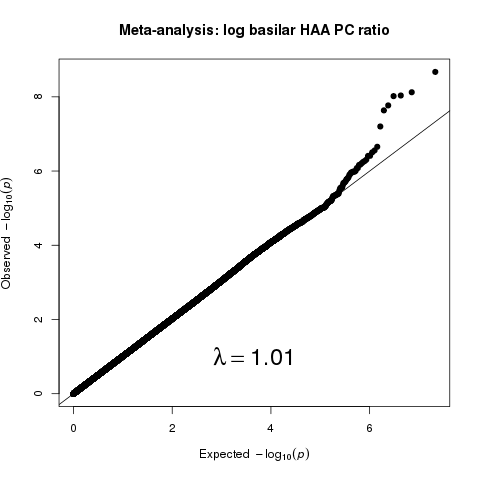
**

**Figure S3: Regional associations plots for statistically significant GWAS regions identified in race/ethnic specific analysis for percent HAA.** (A) Chromosome 4 region including *GNPDA1* among White; (B) Chromosome 6 region including *FOXP4* among White; (C) Chromosome 12 including *ZNF664*-*FAM101A* among White; (D) Chromosome 14 including *DAAM1* among White; (E) Chromosome 10 including *PFKP* among Chinese. All plots for White, African Americans and Chinese are generated using LocusZoom [21] with 1000 Genomes EUR, AFR, and ASN reference panels, respectively, for calculating linkage disequilibrium. Genotyped SNPs are marked as crosses and imputed SNPs are shown as circles.

(A)

**Figure S3 (continued):**

(B)

(C)

**Figure S3 (continued):**

(D)

(E)

**Figure S4: Regional associations plots for statistically significant GWAS regions identified in race/ethnic specific analysis for basilar percent HAA.** (A) Chromosome 1 including *UBE2U* among White; (B) Chromosome 2 including *GYPC* among White; (C) Chromosome 3 including *ALCAM* among White; (D) Chromosome 6 including *STK38* among African American; (E) Chromosome 8 including *FUT10* among African American; (F) Chromosome 14 including *SAMD4A* among African American; (G) Chromosome 10 including *SORCS3* among Chinese. All plots for White, African Americans and Chinese are generated using LocusZoom [21] with 1000 Genomes EUR, AFR, and ASN reference panels, respectively, for calculating linkage disequilibrium. Genotyped SNPs are marked as crosses and imputed SNPs are shown as circles.

(A)

**Figure S4 (continued):**

(B)

(C)

**Figure S4 (continued):**

(D)

(E)

**Figure S4 (continued):**

(F)

(G)

**Figure S5: Regional associations plots for statistically significant GWAS regions identified in race/ethnic specific analysis for basilar HAA peel-core ratio.** (A) and (B) Chromosome 1 including *SLC45A1* and *FAM69A* among White; (C) Chromosome 5 including *GFPT2* among White; (D) Chromosome 18 including *SEH1L* among White; (E) Chromosome 8 including *NCOA2* in African Americans; (F) Chromosome 14 at *SLC25A21* in African Americans. All plots for White, African Americans and Chinese are generated using LocusZoom [21] with 1000 Genomes EUR, AFR, and ASN reference panels, respectively, for calculating linkage disequilibrium. Genotyped SNPs are marked as crosses and imputed SNPs are shown as circles.

(A)

**Figure S5 (continued):**

(B)

(C)

**Figure S5 (continued):**

(D)

(E)

(F)

**References**

1. Bild DE, Bluemke DA, Burke GL, Detrano R, Diez Roux AV, Folsom AR, et al. Multi-ethnic study of atherosclerosis: objectives and design. Am J Epidemiol. 2002; 156:871-881.

2. Kaufman JD, Adar SD, Allen RW, Barr RG, Budoff MJ, Burke GL, et al. Prospective study of particulate air pollution exposures, subclinical atherosclerosis, and clinical cardiovascular disease: The Multi-Ethnic Study of Atherosclerosis and Air Pollution (MESA Air). Am J Epidemiol. 2012; 176:825-837.

3. Carr JJ, Nelson JC, Wong ND, McNitt-Gray M, Arad Y, Jacobs DR, Jr., et al. Calcified coronary artery plaque measurement with cardiac CT in population-based studies: standardized protocol of Multi-Ethnic Study of Atherosclerosis (MESA) and Coronary Artery Risk Development in Young Adults (CARDIA) study. Radiology. 2005; 234:35-43.

4. Tschirren J, McLennan G, Palagyi K, Hoffman EA, Sonka M. Matching and anatomical labeling of human airway tree. IEEE Trans Med Imaging. 2005; 24:1540-1547.

5. Hu S, Hoffman EA, Reinhardt JM. Automatic lung segmentation for accurate quantitation of volumetric X-ray CT images. IEEE Trans Med Imaging. 2001; 20:490-498.

6. Zhang L, Hoffman EA, Reinhardt JM. Atlas-driven lung lobe segmentation in volumetric X-ray CT images. IEEE Trans Med Imaging. 2006; 25:1-16.

7. Guo J RJ, Kitaoka H, Zhang L. Integrated system for CT-based assessment of parenchymal lung disease. In IEEE International Symptosium on Biomedical Imaging; New York. Institute of Electrical and Electronics Engineers; 2002: 871-874.

8. Hoffman EA, Jiang R, Baumhauer H, Brooks MA, Carr JJ, Detrano R, et al. Reproducibility and validity of lung density measures from cardiac CT Scans--The Multi-Ethnic Study of Atherosclerosis (MESA) Lung Study. Acad Radiol. 2009; 16:689-699.

9. Lederer DJ, Enright PL, Kawut SM, Hoffman EA, Hunninghake G, van Beek EJ, et al. Cigarette smoking is associated with subclinical parenchymal lung disease: the Multi-Ethnic Study of Atherosclerosis (MESA)-lung study. Am J Respir Crit Care Med. 2009; 180:407-414.

10. Ferris BG. Epidemiology Standardization Project. Am Rev Respir Dis. 1978; 118 (Supp 2):1-120.

11. Rodriguez J, Jiang R, Johnson WC, MacKenzie BA, Smith LJ, Barr RG. The association of pipe and cigar use with cotinine levels, lung function, and airflow obstruction: a cross-sectional study. Ann Intern Med. 2010; 152:201-210.

12. Pellegrino R, Viegi G, Brusasco V, Crapo RO, Burgos F, Casaburi R, et al. Interpretative strategies for lung function tests. European Respiratory Journal. 2005; 26:948-968.

13. Hankinson JL, Kawut SM, Shahar E, Smith LJ, Stukovsky KH, Barr RG. Performance of American Thoracic Society-recommended spirometry reference values in a multiethnic sample of adults: the multi-ethnic study of atherosclerosis (MESA) lung study. Chest. 2010; 137:138-145.

14. Manichaikul A, Naj AC, Herrington D, Post W, Rich SS, Rodriguez A. Association of SCARB1 Variants With Subclinical Atherosclerosis and Incident Cardiovascular Disease: The Multi-Ethnic Study of Atherosclerosis. Arterioscler Thromb Vasc Biol. 2012.

15. Patterson N, Price AL, Reich D. Population structure and eigenanalysis. PLoS Genet. 2006; 2:e190.

16. Price AL, Patterson NJ, Plenge RM, Weinblatt ME, Shadick NA, Reich D. Principal components analysis corrects for stratification in genome-wide association studies. Nat Genet. 2006; 38:904-909.

17. Li Y, Willer CJ, Ding J, Scheet P, Abecasis GR. MaCH: using sequence and genotype data to estimate haplotypes and unobserved genotypes. Genet Epidemiol. 2010; 34:816-834.

18. Manichaikul A, Mychaleckyj JC, Rich SS, Daly K, Sale M, Chen WM. Robust relationship inference in genome-wide association studies. Bioinformatics. 2010; 26:2867-2873.

19. Marchini J, Howie B, Myers S, McVean G, Donnelly P. A new multipoint method for genome-wide association studies by imputation of genotypes. Nat Genet. 2007; 39:906-913.

20. Chen MH, Yang Q. GWAF: an R package for genome-wide association analyses with family data. Bioinformatics. 2010; 26:580-581.

21. Willer CJ, Li Y, Abecasis GR. METAL: fast and efficient meta-analysis of genomewide association scans. Bioinformatics. 2010; 26:2190-2191.

22. Seibold MA, Wise AL, Speer MC, Steele MP, Brown KK, Loyd JE, et al. A common MUC5B promoter polymorphism and pulmonary fibrosis. N Engl J Med. 2011; 364:1503-1512.

23. Fingerlin TE, Murphy E, Zhang W, Peljto AL, Brown KK, Steele MP, et al. Genome-wide association study identifies multiple susceptibility loci for pulmonary fibrosis. Nat Genet. 2013; 45:613-620.

24. Mushiroda T, Wattanapokayakit S, Takahashi A, Nukiwa T, Kudoh S, Ogura T, et al. A genome-wide association study identifies an association of a common variant in TERT with susceptibility to idiopathic pulmonary fibrosis. J Med Genet. 2008; 45:654-656.

25. Noth I, Zhang Y, Ma SF, Flores C, Barber M, Huang Y, et al. Genetic variants associated with idiopathic pulmonary fibrosis susceptibility and mortality: a genome-wide association study. Lancet Respir Med. 2013; 1:309-317.
